# Artificial intelligence-driven radiomics study in cancer: the role of feature engineering and modeling

**DOI:** 10.1186/s40779-023-00458-8

**Published:** 2023-05-16

**Authors:** Yuan-Peng Zhang, Xin-Yun Zhang, Yu-Ting Cheng, Bing Li, Xin-Zhi Teng, Jiang Zhang, Saikit Lam, Ta Zhou, Zong-Rui Ma, Jia-Bao Sheng, Victor C. W. Tam, Shara W. Y. Lee, Hong Ge, Jing Cai

**Affiliations:** 1 Department of Medical Informatics, Nantong University, Nantong, 226001 Jiangsu China; 2 Department of Health Technology and Informatics, the Hong Kong Polytechnic University, Hong Kong, 999077 China; 3 The Hong Kong Polytechnic University Shenzhen Research Institute, Shenzhen, 518000 Guangdong China; 4 Department of Radiation Oncology, the Affiliated Cancer Hospital of Zhengzhou University and Henan Cancer Hospital, Zhengzhou, 450008 Henan China

**Keywords:** Artificial intelligence, Radiomics, Feature extraction, Feature selection, Modeling, Interpretability, Multi-modalities, Head and neck cancer

## Abstract

Modern medicine is reliant on various medical imaging technologies for non-invasively observing patients’ anatomy. However, the interpretation of medical images can be highly subjective and dependent on the expertise of clinicians. Moreover, some potentially useful quantitative information in medical images, especially that which is not visible to the naked eye, is often ignored during clinical practice. In contrast, radiomics performs high-throughput feature extraction from medical images, which enables quantitative analysis of medical images and prediction of various clinical endpoints. Studies have reported that radiomics exhibits promising performance in diagnosis and predicting treatment responses and prognosis, demonstrating its potential to be a non-invasive auxiliary tool for personalized medicine. However, radiomics remains in a developmental phase as numerous technical challenges have yet to be solved, especially in feature engineering and statistical modeling. In this review, we introduce the current utility of radiomics by summarizing research on its application in the diagnosis, prognosis, and prediction of treatment responses in patients with cancer. We focus on machine learning approaches, for feature extraction and selection during feature engineering and for imbalanced datasets and multi-modality fusion during statistical modeling. Furthermore, we introduce the stability, reproducibility, and interpretability of features, and the generalizability and interpretability of models. Finally, we offer possible solutions to current challenges in radiomics research.

## Background

Cancer is a devastating disease that affects many people worldwide [1]. Cancerous tumors start as a small cluster of neoplastic cells that may be located within an intricate network of internal tissues and organs, which makes it difficult to diagnose such cancers (e.g., nasopharyngeal carcinoma) in their early stages [2]. In addition, cancers of the same type and stage may behave remarkably differently in different patients, so it is critical that methods are available to monitor tumor growth, to assist clinicians in prescribing anti-cancer treatment, and to assess treatment responses in individual patients [3].

In this regard, medical imaging, such as computed tomography (CT), magnetic resonance imaging (MRI), positron emission tomography (PET), and ultrasonography (US), is indispensable for detecting the presence and monitoring the growth of cancer, and assessing treatment responses. Different imaging modalities capture different properties of internal organs. For instance, CT detects anatomical changes in organs, such as arterial calcification [4]; MRI visualizes soft-tissue contrast and the musculoskeletal system [5]; and PET captures functional and metabolic changes in tissues or organs [6]. Contrast agents are often employed to enhance visualization of the contrast between signal intensities in images of normal and abnormal tissues (such as tumors). Nonetheless, clinical judgement based on unaided visual inspection of images can be resource intensive, is dependent on physicians’ experience, and may fail to detect all of the information within the three-dimensional (3D) volume of a tumor.

Radiomics has recently emerged as a promising solution to these problems, as it involves high-throughput extraction and analysis of high-dimensional quantitative features from multi-modal medical images [7], which enables it to non-invasively capture intratumoral heterogeneity [8]. Radiomics-based studies consist of the following six steps: image acquisition, image preprocessing, image segmentation, feature extraction, feature selection, and model construction and evaluation [9]. The key steps are those involved in feature engineering (i.e., feature extraction and feature selection) and statistical modeling (i.e., model construction and evaluation) and are the current focus of most researchers’ efforts. Moreover, good progress in feature engineering and statistical modeling has been made in recent years. For example, radiomics features are now known to be correlated with tumor diagnosis and prognosis, so researchers have used the minimal redundancy maximal relevance (mRMR) method, the least absolute shrinkage and selection operator (LASSO), and other technologies to select predictive radiomics features. They have also used classifiers such as support-vector machine (SVM) and random forest (RF) to construct radiomics-based models. Numerous studies have also constructed radiomics-based models to, for example, aid in cancer diagnosis, prognosis, and the prediction of treatment responses. These models have demonstrated the possibility of developing risk stratification and personalized treatment for patients, which could lead to the realization of precision medicine. However, despite this progress in radiomics, several key problems remain to be solved.

In this review, we summarize recent literature on applications of radiomics for the investigation of tumors, with a particular focus on feature engineering and statistical modeling methods. We also review aspects that may influence model performance, such as feature stability and model generalizability; highlight the problems that remain to be comprehensively solved (such as imbalanced datasets and multi-modality fusion); and make recommendations to the community for future research and development.

## Clinical applications based on radiomics

### Radiomics-based cancer diagnosis

Traditional medical imaging plays an important role in the diagnosis of cancer, but misdiagnoses and missed diagnoses nevertheless occur. These are major problems, as they prevent early diagnoses and thus timely clinical intervention, thereby decreasing cancer patients’ survival rates and cure rates [10, 11]. This problem can potentially be addressed by the augmentation of traditional medical imaging with radiomics, which can capture phenotypic information of tumors [12] and has shown promise in differentiating benign and malignant tumors and predicting treatment responses. Radiomics analysis relies on artificial intelligence (AI) algorithms, which can improve the accuracy (Acc) of predictive models used for the diagnosis and evaluation of treatment responses. In particular, radiomics applies feature engineering to detect intra-tumoral properties in medical images that are typically undetected during visual inspection of such images by physicians. In the following, we provide a comprehensive overview of the utilization of radiomics-based methods for cancer diagnosis, with a particular focus on three distinct perspectives: tumor grading, tumor staging, and the classification of malignant vs. benign tumors.

#### Radiomics-based tumor grading

Reliable pre-surgical radiomics-based evaluation of tumor grading can help to formulate treatment plans for patients and can also reduce the recurrence rate and incidence of adverse effects. As shown in Table 1 [13–16], radiomics analysis has been explored for tumor grading (i.e., describing the magnitude of tumor atypia) in various cancers like head and neck cancers (HNCs) and lung cancers, as it is an efficient non-invasive method for pathological examination. Specifically, Wu et al. [13] constructed a radiomics signature with kernel principal component analysis (KPCA), a RF classifier, and a variance-threshold, which they used to develop a radiomics model. They then compared the performance of this model against that of a clinical model and a combined clinical-radiomics model in the analysis of CT images for the grading of head and neck squamous cell carcinoma (HNSCC) tumors. They found that the combined model outperformed the other two models, as their respective areas under the receiver operating characteristic curve (AUCs) were 0.97, 0.96 and 0.63. Mukherjee et al. [14] used CT-based radiomics features to develop models for HNSCC tumor grading, predicting perineural invasion, and lymphovascular invasion, and these models’ AUCs were 0.66, 0.70, and 0.65, respectively. Although these performances suggest that these models are unsuitable for clinical adoption, they may be sufficient to demonstrate the potential of CT-based radiomics features for predicting histopathologic characteristics.

**Table 1:** Applications of radiomics-based tumor grading

Image modality	Number of patients	Cancer	Target	Number of radiomics features	Commercial or open-source software	Method	References
CT	206	HNSCC	Tumor grading	74	Matlab, Python, IBM SPSS software	ML: KPCA, RF, VT selection SM: DeLong test, *t*-test, Chi-square test	[13]
CT	284	HNSCC	Tumor grading, extracapsular spread, perineural invasion, lymphovascular invasion, human papillomavirus status	25–35	Matlab, R	ML: PCA, LR, LASSO, Hierarchic clustering, tenfold CV SM: Fisher exact test	[14]
CT	878	Lung cancer, HNC	Tumor grading	Unspecified	Matlab, R	ML: LR, consensus clustering, hierarchical clustering SM: Jaccard index, Pearson correlation analysis	[15]
CT	211	Laryngeal cancer	Preoperative T category (T_3_ vs. T_4_)	8	ITK-SNAP, PyRadiomics, R, Python	ML: LASSO, SVM, Grid search, CV SM: *t*-test (or Mann–Whitney *U* test), Chi-square (or Fisher’s exact) test, ICC	[16]

*CT* computed tomography, *ML* machine learning, *SM* statistical method, *HNSCC* head and neck squamous cell carcinoma, *HNC* head and neck cancer, *KPCA* kernel principal component analysis, *RF* random forest, *VT* variance-threshold, *PCA* principal component analysis, *LR* logistic regression, *LASSO* least absolute shrinkage and selection operator, *CV* cross validation, *SVM* support vector machine, *ICC* intraclass correlation coefficients

#### Radiomics-based tumor staging

Some studies have focused on developing radiomics-based models for tumor staging as shown in Table 2 [17–20], which classifies the severity of a malignancy according to the size of the primary tumor and the extent of its spread throughout the body. Traditionally, tumor staging information is obtained by performing imaging examinations and pathological biopsies; in contrast, radiomics can be applied for preoperative tumor staging and thus may be more effective. For example, we included three radiomics studies [17–19] that addressed preoperative tumor staging of different cancers. Ren et al. [17] preoperatively distinguished I–II and III–IV stage HNSCC tumors by analyzing the radiomics features of T2-weighted (T2W) and contrast-enhanced T1-weighted (ceT1W) MR images. They found that the radiomics signatures based on the ceT1W images (AUC = 0.853) best discriminated between stage I–II and stage III–IV HNSCC tumors, followed by models based on T2W and ceT1W combined images (AUC = 0.849). In the study by Gao et al. [20], a radiomics model based on 30 US features was constructed to evaluate tumor staging. The study employed ten classifiers and observed that the Naive Bayes model attained an AUC of 0.84 in the validation cohort. Furthermore, tumor staging can be performed using CT imaging, MR imaging, and US imaging. It is notable that CT imaging is a less time-consuming and less costly option compared to MR imaging; however, MR imaging is superior in distinguishing soft tissue changes from cartilage abnormalities. Notably, US imaging is less expensive than both CT and MR imaging, but its resolution is inferior to that of CT imaging. Therefore, an appropriate imaging mode should be selected according to the research objectives of a given situation.

**Table 2:** Applications of radiomics-based tumor staging

Image modality	Number of patients	Cancer	Target	Number of radiomics features	Commercial or open-source software	Method	References
MRI	127	HNSCC	Preoperative staging (stage I–II from stage III–IV)	6	ITK-SNAP, Matlab, R, SPSS	ML: LASSO, LR SM: Mann–Whitney *U* test, McNemar test	[17]
CT	154	Esophageal cancer	Preoperative staging	10	Matlab, R	ML: LASSO, fivefold CV SM: Mann–Whitney *U* test, DeLong test, Net reclassification improvement, Chi-square test, ICC	[18]
CT	494	Primary colorectal cancer	Preoperative staging	16	Matkab, SPSS	ML: LASSO, LR SM: Mann–Whitney *U* test, DeLong test	[19]
US	157	Bladder cancer	Tumor staging	30	ITK-SNAP, Intelligence Foundry, SPSS	ML: SVM-RFE, L1-regularized LR, Random forests, DT, Naive Bayes, KNN, Bagging, Extremely RF, AdaBoost, Gradient boosting regression trees, fivefold CV SM: *t*-test, Chi-square test, Z-score, Spearman correlation analysis, Mann–Whitney *U* test	[20]

*MRI* magnetic resonance imaging, *CT* computed tomography, *US* ultrasonography, *ML* machine learning, *SM* statistical method, *HNSCC* head and neck squamous cell carcinoma, *LASSO* least absolute shrinkage and selection operator, *LR* logistic regression, *CV* cross validation, *ICC* intraclass correlation coefficients, *SVM* support vector machine, *RFE* recursive feature elimination, *DT* decision tree, *KNN* K-nearest neighbors, *RF* random forest, *AdaBoost* adaptive boosting

#### Radiomics-based classification of malignant vs. benign tumors

Pathological examination is the gold-standard approach for the diagnosis of benign and malignant tumors. However, it is an invasive approach, and radiomics can serve as a non-invasive alternative. Table 3 [21–23] summarizes the application of radiomics-based classification of benign and malignant tumors. Ho et al. [21] identified 89 features in MR images that can be used to discriminate between benign and malignant lymph nodes. The most discriminating of these 89 features is “Original_glcm_DifferenceAverage”, which measures the relationship between occurrences of pairs with similar intensity values and occurrences of pairs with different intensity values. In order to identify benign/malignant liver tumors, Yin et al. [22] developed a CT-based radiomics model, which achieved an average AUC of 0.87.

**Table 3:** Applications of radiomics-based classification of malignant versus benign tumors

Image modality	Number of patients	Cancer	Target	Number of radiomics features	Commercial or open-source software	Method	References
MRI	130	HNSCC	Classify benign and malignant tumors, differentiate ENE	89/6	3D Slicer, Segmentation Wizard, Python	ML: Adam optimization algorithm SM: *t*-test DL: Multilayer perceptron neural network	[21]
CT	285	HCC and hepatic hemangioma	Classify benign and malignant tumors	13	Matlab	ML: LR, LASSO, SVM, Multiple-regression	[22]
MRI	69	Parotid lesions	Classify benign and malignant tumors	4	Matlab, S-IBEX	ML: SVM, NCA, CV SM: Chi-square test, Mann–Whitney test, Spearman correlation coefficient, Z-score	[23]

*MRI* magnetic resonance imaging, *CT* computed tomography, *ML* machine learning, *SM* statistical method, *DL* deep learning, *HNSCC* head and neck squamous cell carcinoma, *HCC* hepatocellular carcinoma, *ENE* extra-nodal extension, *LR* logistic regression, *LASSO* least absolute shrinkage and selection operator, *SVM* support vector machine, *NCA* neighborhood component analysis, *CV* cross validation

The above-described studies have accumulated evidence that radiomics analysis has the potential to serve as a non-invasive diagnostic tool prior to the treatment of cancer. In particular, radiomics analysis can increase the Acc and the objectivity of tumor staging and grading, thereby serving as an auxiliary decision tool for personalizing treatment.

### Radiomics-based cancer prognosis prediction

Radiomics may be a better predictor of treatment response, survival, tumor recurrence, or metastasis than other conventional methods [24–27]. Therefore, radiomics could assist clinicians to accurately stratify the negative prognosis risks of patients before treatment, select appropriate treatment plans, support the development of personalized medicine, and thereby substantially improve cancer prognosis. Here, we review the applications of radiomics-based prognosis prediction from the aspects of survival, local recurrence, and metastasis.

#### Radiomics-based survival prediction

Table 4 [15, 28–35] summarizes recent radiomics-based survival studies that have investigated aspects such as death prognosis, all-cause mortality progression-free survival (PFS), and disease-free survival (DFS). Fh et al. [28] developed deep learning models based on radiomics features from planning target volumes (PTVs) and gross tumor volumes (GTVs) to simultaneously predict patients’ death and tumor recurrence. Their model based on GTV radiomics features predicted death and tumor recurrence with Accs of 85.9% and 72.4%, respectively, and overall AUCs of 0.947 and 0.956, respectively, whereas their model based on PTV features predicted these two endpoints with Accs of 77.7% and 74.3%, respectively, and overall AUCs of 0.934 and 0.932, respectively. A 2020 study [30] predicted the PFS time and overall survival (OS) time of oropharyngeal squamous cell carcinoma (OPSCC) by using the American Joint Committee on Cancer (AJCC) staging model [which the AJCC built based on their staging scheme for human papillomavirus (HPV)-related and -unrelated OPSCC] as the baseline model and constructing a PET/CT-based radiomics model. This study found that the average concordance index ± standard deviation of its radiomics model was generally higher than that of the AJCC model in both HPV-related and -unrelated cohorts, which proved that PET/CT radiomics features can add prognostic value beyond that offered by the AJCC staging scheme. Studies [31–35] have also developed radiomics models to predict aspects such as DFS, OS, long-term survival, and recurrence-free survival, further underscoring the utility of radiomics features for predicting cancer survival.

**Table 4:** Applications of radiomics-based survival prediction

Image modality	Number of patients	Cancer	Target	Number of radiomics features	Commercial or open-source software	Method	References
CT	878	Lung cancer and HNSCC	Patient survival	Unspecified	Matlab, R	ML: LR, Consensus clustering, Hierarchical clustering SM: Jaccard index, Pearson correlation analysis	[15]
CT	188	HNSCC	The death prognosis	107	PyRadiomics, 3D Slicer, Matlab	ML: LOOCV SM: Chi-square test DL: Deep learning artificial neural networks	[28]
FDG-PET	174	OPC	The risk of ACM	2–3	Matlab, Stata/MP	ML: LOOCV, Cox proportional-hazards regression, Fine and Gray’s proportional sub-hazards model, LR, fivefold CV SM: Kaplan–Meier analysis, log-rank test, Spearman correlation analysis	[29]
PET, CT, PET/CT	311	Oropharyngeal squamous cell carcinoma	PFS, OS	Unspecified	3D Slicer, PyRadiomics, R, ggplot2	ML: Random survival forest, Threefold stratified CV SM: *t*-test, Kaplan–Meier analysis, log-rank test, C-index	[30]
CT	44	Laryngeal and hypo-pharyngeal cancers	DFS	26	Perfusion-4, ROCKIT	ML: Two-loop leave-one-out, Linear discriminant analysis SM: *t*-test, ICC, Kappa analysis	[31]
MRI	136	EBV-related NPC	OS	2	Matlab, 3D Slicer, PyRadiomics	ML: Cox regression model, tenfold CV SM: Kaplan–Meier analysis, log-rank test, Mann–Whitney test or Spearman correlation analysis, ICC	[32]
MRI	504	NPC	Long-term survival	17	AccuContour, PyRadiomics, X-tile, R	ML: LASSO, Cox regression model, tenfold CV SM: Mann–Whitney *U* test or *t-*test, Kaplan–Meier analyses, log-rank test, Hosmer–Lemeshow test, C-index	[33]
MRI	236	Tongue cancer	DFS, OS	15/17/18/25/10	ITK-SNAP, AIMT, Python, R, SPSS	ML: PCA, SVM, Cox regression analysis, fivefold CV SM: DeLong test, Spearman correlation analysis, Kaplan–Meier analysis, log-rank test, ICC	[34]
MRI	346	Rectal cancer	3-year recurrence-free survival	4/5/10	GE Healthcare, 3D Slicer, R, SPSS	ML: LASSO, LR, Cox analysis SM: ICC, Wilcoxon test, Hosmer–Lemeshow test, *t*-test, Nonparametric test, Chi-square test and Fisher’s exact test, DeLong test	[35]

*CT* computed tomography, *MRI* magnetic resonance imaging, *FDG* fluorodeoxyglucose, *PET* positron emission tomography, *ML* machine learning, *SM* statistical method, *DL* deep learning, *HNSCC* head and neck squamous cell carcinoma, *OPC* oropharyngeal cancer, *NPC* nasopharyngeal carcinoma, *ACM* all-cause mortality, *PFS* progression-free survival, *OS* overall survival, *DFS* disease-free survival, *LR* logistic regression, *LOOCV* leave one out cross validation, *CV* cross validation, *ICC* intraclass correlation coefficients, *LASSO* least absolute shrinkage and selection operator, *PCA* principal component analysis, *SVM* support vector machine

Other studies have focused on determining whether associations exist between selected radiomics features and specific outcomes of patients following chemoradiotherapy. Selecting such features before modeling helps to reduce redundancy and improve the predictive performance of models. Parmar et al. [15] investigated the prognostic features of lung cancer and HNC by applying consensus clustering to generate 11 and 13 radiomics feature clusters of lung cancer and HNC, respectively, and then located the clusters that were highly correlated with cancer prognosis. Subsequently, they constructed models and evaluated their prognostic performance, which revealed that the models generated inconsistent prognoses for the two diseases because the clusters were site specific.

#### Radiomics-based recurrence prediction

Folkert et al. [29] constructed a multivariable predictive model of tumor-related endpoints (all-cause mortality, local failure and distant metastasis) by integrating clinical parameters and fluorodeoxyglucose (FDG)-PET-based radiomics features; this model revealed that metabolic tumor volume (MTV) was correlated with all three endpoints. However, in an independent cohort validation, the multivariable model with local failure (local recurrence) as the endpoint had the highest AUC [0.73 (*P* = 0.026)] and was thus the most predictive; in contrast, the models with all-cause mortality and distant metastasis as endpoints, respectively, had lower AUCs [0.65 (*P* = 0.004) and 0.66 (*P* = 0.015), respectively] and were moderately predictive. There are also radiomics studies about cancer recurrence, which are summarized together with the above study in Table 5 [28, 29, 36–38].

**Table 5:** Applications of radiomics-based recurrence prediction

Image modality	Number of patients	Cancer	Target	Number of radiomics features	Commercial or open-source software	Method	References
CT	188	HNSCC	Cancer recurrence rate	107	PyRadiomics, 3D Slicer, Matlab	ML: LOOCV SM: Chi-square test DL: Deep learning artificial neural networks	[28]
FDG-PET	174	OPC	The risk of local failure	2–3	Matlab, Stata/MP	ML: LOOCV, Cox proportional-hazards regression, Fine and Gray’s proportional sub-hazards model, LR, fivefold CV SM: Kaplan–Meier analysis, log-rank test, Spearman correlation analysis	[29]
CT	465	OPC	Local recurrence	2	Matlab	ML: Bootstrap resampled recursive partitioning analysis, Regression model, DT, Cox proportional hazards model SM: Log-rank and Wilcoxon test, Effect likelihood ratio test, Wald test	[36]
MRI	285	HNSCC	Local tumor recurrence	20	MITK, SPM, Matlab, R	ML: LASSO, tenfold CV SM: *t*-test, Chi-square test or Fisher’s exact test, Delong test, Spearman correlation analysis	[37]
US	83	Breast cancer	Recurrence	4	Matlab, SPSS	ML: KNN, SVM SM: Shapiro–Wilk test, *t*-test, Mann–Whitney test, Kaplan–Meier product-limit method	[38]

*CT* computed tomography, *MRI* magnetic resonance imaging, *FDG* fluorodeoxyglucose, *PET* positron emission tomography, *US* ultrasonography, *ML* machine learning, *SM* statistical method, *DL* deep learning, *HNSCC* head and neck squamous cell carcinoma, *OPC* oropharyngeal cancer, *LOOCV* leave one out cross validation, *LR* logistic regression, *CV* cross validation, *DT* decision tree, *LASSO* least absolute shrinkage and selection operator, *KNN* K-nearest neighbors, *SVM* support vector machine

#### Radiomics-based metastasis prediction

Regarding distant metastasis of cancer, one study [39] investigated a distant metastasis model based on MRI features, extracting a total of 2803 radiomics features from the MR images of 176 patients. They then screened these features to yield seven features that were used with a logistic regression algorithm to form a distant metastasis model that was superior to a clinical factor-based model. Subsequently, they constructed a nomogram that can help to determine the risk of metastasis for a patient and develop personalized treatment plans.

Lymph node metastasis (LNM) is traditionally adopted to guide decision-making on prescribed treatments for cancer [40]. The occurrence of LNM usually indicates a poor prognosis, so it is essential to count in LNM when treating tumors. Wang et al. [34] extracted radiomics features from T2W MR images of primary tumors with and without 3D peritumoral extensions (3, 5, 10, and 15 mm, respectively) and incorporated these features with clinicopathological features. They then constructed combined models using an SVM classifier and found that with the test set, the combined model based on 10‐mm peritumoral extensions achieved a higher AUC (0.872) in predicting LNM than the combined models based on no peritumoral extensions (AUC = 0.720), 3-mm peritumoral extensions (AUC = 0.787), 5-mm peritumoral extensions (AUC = 0.793), and 15-mm peritumoral extensions (AUC = 0.808). A study published in 2020 [41] analyzed the relationship between radiomics features extracted from US images and LNM of thyroid cancer. This showed that only an irregular shape and microcalcification were effective predictors of LNM, with AUCs of 0.591 (*P* = 0.059) and 0.629 (*P* = 0.007), respectively. Table 6 [29, 34, 35, 39, 41–43] shows the main content of recent radiomics studies of cancer metastasis.

**Table 6:** Applications of radiomics-based metastasis prediction

Image modality	Number of patients	Cancer	Target	Number of radiomics features	Commercial or open-source software	Method	References
FDG-PET	174	OPC	The risk of DM	2–3	Matlab, Stata/MP	ML: LOOCV, Cox proportional-hazards regression, Fine and Gray’s proportional sub-hazards model, LR, fivefold CV SM: Kaplan–Meier analysis, log-rank test, Spearman correlation analysis	[29]
MRI	176	NPC	DM	7	PyRadiomics, Python, ITK-SNAP, R, SPSS	ML: mRMR, LASSO, LR, Mutual information, Bootstrap-resampling SM: ICC, *t*-test, Kaplan–Meier analysis, log-rank test, Fisher's exact test, Chi-square test, or Mann–Whitney *U* test	[39]
MRI	236	Tongue cancer	LNM	15/17/18/25/10	ITK-SNAP, AIMT, Python, R, SPSS	ML: PCA, SVM, Cox regression analysis, fivefold CV SM: DeLong test, Spearman correlation analysis, Kaplan–Meier analysis, log-rank test	[34]
MRI	346	Rectal cancer	LNM	4/5/10	GE Healthcare, 3D Slicer, R, SPSS	ML: LASSO, LR, Cox analysis SM: ICC, Wilcoxon test, Hosmer–Lemeshow test, *t*-test, Nonparametric test, Chi-square test, and Fisher’s exact test, DeLong test	[35]
US	126	Thyroid cancer	LNM	91	ITK-SNAP, Ultrosomics, SPSS	ML: LASSO, PCA, DT, Naive Bayes, KNN, LR, SVM, Bagging, RF, Extremely RF, AdaBoost, Gradient boosting DT SM: *t*-test, Chi-square test or Fisher’s exact test	[41]
US	205	NPC	LNM	7	GE Healthcare, R, Python	ML: mRMR, LR, LASSO SM: ICC, DeLong test	[42]
PET	76	Primary prostate cancer	LNM, DM	22	RaCaT, Python	ML: RF, CV, PCA SM: Chi-square test, DeLong test, ICC, Z-score	[43]

*CT* computed tomography, *MRI* magnetic resonance imaging, *FDG* fluorodeoxyglucose, *PET* positron emission tomography, *US* ultrasonography, *ML* machine learning, *SM* statistical method, *OPC* oropharyngeal cancer, *NPC* nasopharyngeal carcinoma, *DM* distant metastasis, *LNM* Lymph node metastasis, *LOOCV* leave one out cross validation, *LR* logistic regression, *CV* cross validation, *mRMR* maximum relevance minimum redundancy, *LASSO* least absolute shrinkage and selection operator, *ICC* intraclass correlation coefficients, *PCA* principal component analysis, *SVM* support vector machine, *DT* decision tree, *KNN* K-nearest neighbors, *RF* random forest, *AdaBoost* adaptive boosting

### Radiomics-based cancer treatment responses prediction

As treatment responses are closely related to OS, seven studies [44–50] have investigated the application of radiomics for constructing models to predict treatment responses to radical radiotherapy, chemotherapy or chemoradiotherapy. For example, one of these studies [44] used quantitative US (QUS) delta-radiomics to monitor the response of HNC to radical radiotherapy. K-nearest neighbors (KNN) and Naive Bayes algorithms were both used to construct single-, two-, and three-feature models. The results showed that the single-feature Naive Bayes model had the highest Acc in predicting responses after 3 months of treatment: its Acc based on the QUS characteristics at 24 h after chemoradiotherapy was 80%, and increased to 85% when the QUS characteristics obtained at the fourth week were included.

In addition, a dual-center retrospective study [47] was performed to extract radiomics features from the MR images of 221 patients before induction chemotherapy (IC) and 96 patients after IC, and then calculate the delta-radiomics feature values (by subtracting the feature values from MR images obtained after IC from those obtained before IC) and process them. Subsequently, a pre-treatment MRI radiomics model and a delta-radiomics model were generated and trained using pre-treatment MRI radiomics features and delta-radiomics features, respectively, to predict the tumor retraction response to IC plus concurrent chemoradiotherapy. The trained models were then applied to an external validation set and afforded AUCs of 0.983 and 0.818, respectively, demonstrating their potential utility as references for devising cancer-treatment plans. Table 7 [44–50] summarizes recent radiomics-based treatment response studies.

**Table 7:** Applications of radiomics-based treatment response prediction

Image modality	Number of patients	Cancer	Target	Number of radiomics features	Commercial or open-source software	Method	References
US	36	HNC	Responses to radical radiotherapy	1–3	SPSS, Matlab	ML: Naïve Bayes, KNN, Leave-one-out CV SM: Shapiro–Wilk test,* t*-test, Mann–Whitney U-test, Kaplan–Meier analysis, log-rank test	[44]
CT	290	HNSCC	Incomplete response to definitive radiotherapy or chemo-radiation	Unspecified	PyRadiomics, Python	ML: L1-penalized (LASSO) LR, MI, Grid search with CV, fivefold CV SM: F-test, ANOVA, Pearson correlation analysis	[45]
CT	27	HNSCC	Lymph node response to IC	3	3D Slicer, R	ML: LASSO, LR, fivefold CV SM: Pearson correlation analysis	[46]
MRI	272	NPC	Tumor retraction to IC combined with concurrent chemo-radiotherapy	7/12	ITK-SNAP, Artificial Intelligence Kit, R	ML: mRMR, LASSO, LR, tenfold CV SM: ICC, Z-score	[47]
MRI	137	Rectal cancer	Treatment response to NAC	19	ITK-SNAP, Python, R	ML: LASSO, LR SM: ICC, Pearson correlation analysis, Univariate analysis, Backward elimination, Chi-square test or Fisher’s exact test, the Kruskal–Wallis test	[48]
MRI	140	Breast cancer	Pathologic complete response to NAC	5	ITK-SNAP, GE Healthcare, Python, R	ML: LASSO, LR, fivefold CV SM: ICC, ANOVA, *t*-test, Spearman correlation analysis. Mann–Whitney *U* test, Chi-square test or Fisher’s exact test, Hosmer–Lemeshow test, DeLong test	[49]
MRI	634	Rectal cancer	T downstaging (ypT0-2) after NAC	Unspecified	SPSS, Precision Medicine Open Platform, R, SIMCA	ML: PCA, SVM, LR, LASSO, Partial least-squares discriminant analysis, RF SM: Mann–Whitney U test, Fisher’s exact test, Univariate analyses, Multivariate analyses, Pearson correlation analysis, ANOVA	[50]

*CT* computed tomography, *MRI* magnetic resonance imaging, *US* ultrasonography, *ML* machine learning, *SM* statistical method, *HNC* head and neck cancer, *HNSCC* head and neck squamous cell carcinoma, *NPC* nasopharyngeal carcinoma, *IC* induction chemotherapy, *NAC* neoadjuvant chemotherapy, *KNN* K-nearest neighbors, *CV* cross validation, *LR* logistic regression, *MI* mutual information, *ANOVA* analysis of variance, *LASSO* least absolute shrinkage and selection operator, *mRMR* maximum relevance minimum redundancy, *ICC* intraclass correlation coefficients, *PCA* principal component analysis, *SVM* support vector machine, *RF* random forest

### Radiomics-based cancer treatment complications prediction

Radiation toxicity is an important consideration for treatment optimization, and its accurate prediction allows enhanced personalization of treatment plans. Many studies have investigated adverse effects of radiotherapy and chemotherapy on tissues. For instance, radiotherapy for nasopharyngeal carcinoma may cause cervical spine osteoradionecrosis [51], which is difficult to distinguish from bone metastasis by visual inspection of images. However, radiomics was demonstrated to have great potential for the accurate detection of cervical spine osteoradionecrosis. For example, Zhong et al. [52] used a LASSO logistic regression algorithm based on tenfold cross-validation of the minimum criteria to select eight relevant features, which they used to develop a radiomics nomogram that can distinguish osteoradionecrosis from cancer bone metastasis. The AUC of this nomogram reached 0.72 in the validation cohorts. Xerostomia is the most common side-effect of radiotherapy for HNC, and its prediction based on radiomics features has been extensively investigated. In a study published in 2018 [53], the lesion regions of interest (ROIs) on MR images of parotid glands were delineated using the target contour of CT images. Then, a reference model that predicts xerostomia based only on the parotid gland radiotherapy dose and patient-reported xerostomia at the start of radiotherapy was fitted. The reference model had an AUC in the external validation of 0.65, which was increased to 0.83 by the addition of quantified MRI features, thereby improving its ability to predict the occurrence of xerostomia.

Most of the radiomics studies conducted to date have been retrospective, which can lead to overestimation of the performance of radiomics models. In contrast, applying these models to prospective studies requires a sufficiently large training cohort and strong performance. Prospective studies also involve recruiting participants in advance without relying on existing patients’ data, and conducting long-term follow-ups to evaluate the results predicted by the model. While the results of these studies cannot be communicated to clinicians and participants, they provide a more reliable estimate of model performance. The excellent results obtained in these studies demonstrate the high generalizability and robustness of radiomics, making it a viable tool for clinical applications. The six prospective studies we reviewed [35, 38, 43, 48–50] showed acceptable performance of the radiomics model, with AUCs ranging from 0.688 to 0.871. Although the lowest AUC value of 0.688 [35] suggests that the model has limited classification performance, it still demonstrates predictive power. The study with the highest AUC value of 0.871 [48] also had an acceptable number of participants in the prospective cohort.

## AI-driven radiomics studies

**Fig. 1:**
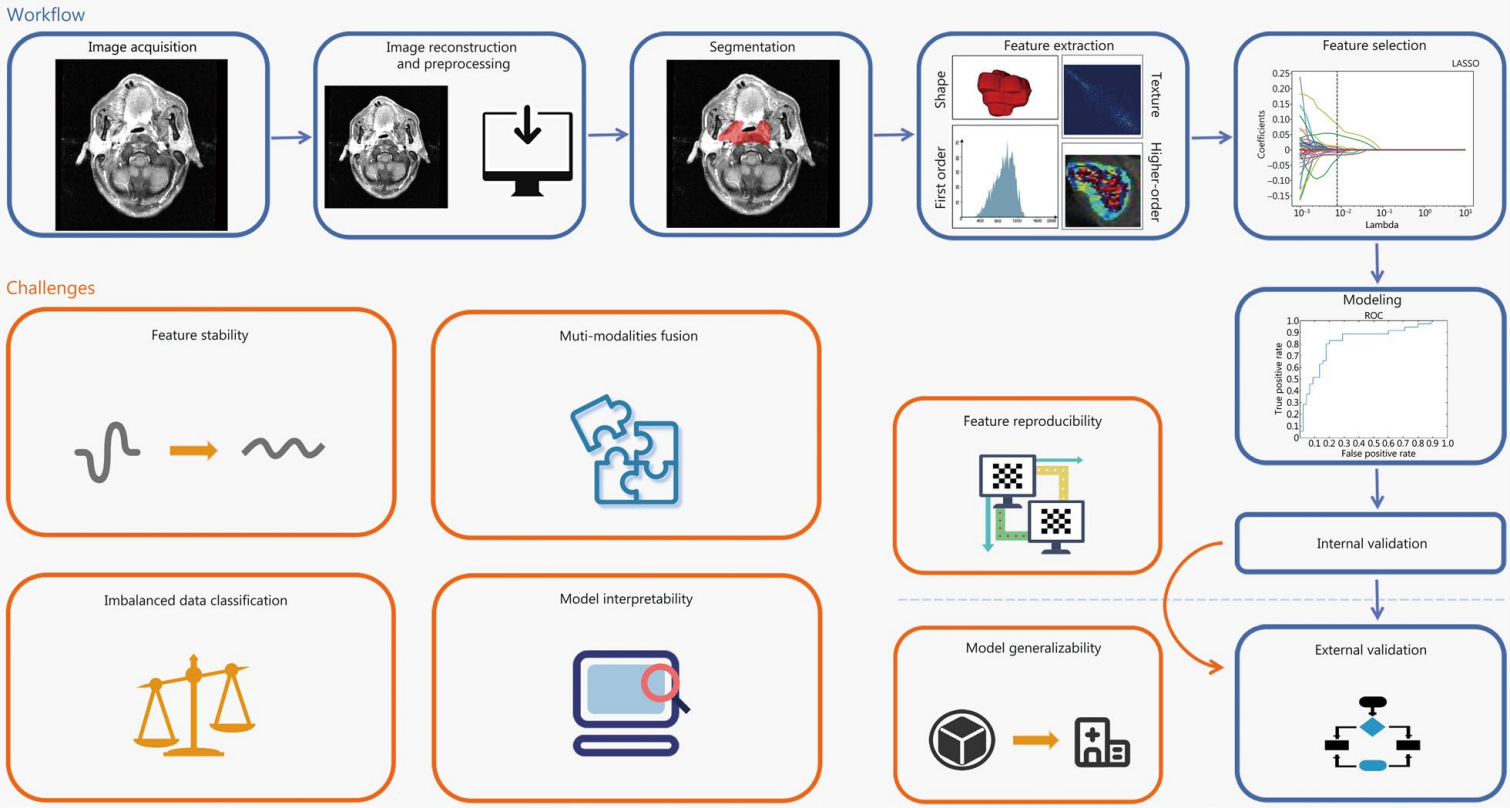
Workflow and challenges of radiomics-based studies. The workflow of radiomics involves several key stages including image acquisition, image reconstruction and preprocessing, image segmentation, feature extraction and selection, model construction, and internal and external validation. However, there are still several challenges that need to be addressed in this workflow such as ensuring feature stability and reproducibility, improving model generalizability and interpretability, addressing imbalanced data classification, and improving multi-modality fusion in statistical modeling. It is worth noting that feature engineering and statistical modeling are two important components of radiomics

The workflow and challenges of radiomics-based studies are illustrated in Fig. 1, based on which we discuss feature engineering and statistical modeling. Feature engineering focuses on the reproducibility, and interpretability of features, whereas statistical modeling focuses on the generalizability of a given model, imbalanced data classification, multi-modality fusion of the model, and interpretability of the model. In addition, feature reproducibility is one of the factors influencing model generalizability. Thus, an improvement in feature reproducibility can enhance model generalizability, which means that the model can be popularized better in clinical practice.

In this section, we review studies that have offered insights into and suggestions on how to solve feature engineering and statistical modeling challenges in radiomics.

### Feature engineering

#### Feature extraction

Feature extraction involves comprehensively quantifying the tumor phenotypes based on high-throughput features that are hypothetically associated with the tumor microenvironment. Specifically, after completion of some steps of the radiomics workflow defined above, such as image acquisition, preprocessing, and segmentation, radiomics features associated with a given research purpose are extracted from within two-dimensional (2D) or 3D ROIs in images. The selection of ROIs is diverse. The majority of radiomics studies discussed non-metastatic carcinoma (M0) population, so the primary tumor generally is the ROI for feature extraction. On the other hand, there are a few radiomics articles that studied the metastatic population (M1) [54], therefore it is possible for them to select the metastasis site as the ROI for feature extraction. In addition, the intra-tumoral region and the peritumoral region can also be ROI for feature extraction, and the features of the peritumor region may show better prognostic performance [55]. Therefore, the selection of ROIs primarily depends on the study population.

**Fig. 2:**
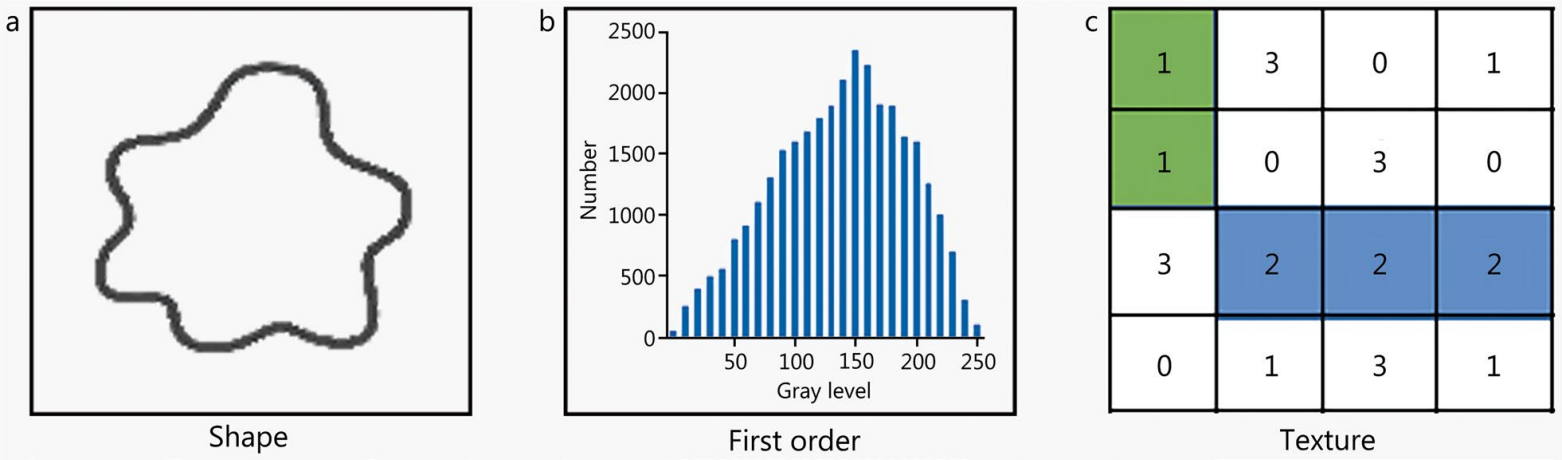
Categories of radiomics features. Radiomics features including shape features, first order features and texture features. **a** Shape features describe the shape of ROI such as sphericity and volume. **b** First order features, such as mean value, median value and entropy, are calculated based on the histogram to describe the distribution of individual voxels values, regardless of the spatial relationship. **c** Texture features quantify the spatial relationship between voxels, which obtained from various matrix types, such as gray level co-occurrence matrix features, gray level run length matrix features, gray level dependence matrix features, gray level size zone matrix features, and neighbouring gray-tone difference matrix features

These are generally distinctive types of radiomics features of ROIs, such as shape, first-order, and texture features (Fig. 2). Shape features define the shape of an ROI, such as its sphericity, volume, and surface area. First order features describe the properties of histogram, regardless of the spatial relationship [56]. such as mean value, median value and entropy. Texture features describe the properties of secondary matrix, such as gray level co-occurrence matrix features [57], gray level run-length matrix features [58], gray level dependence matrix features [59], gray level size zone matrix features, and neighboring gray tone difference matrix features. It follows that textural features can quantify the spatial relationships between voxels. Furthermore, radiomics features can be extracted from original images, log-sigma-filtered images, and wavelet-filtered images, with those extracted from the latter two types of images generally referred to as higher-order features.

Generally, 2D features are extracted from single-layer images containing the most typical or largest cross-section of a lesion, whereas 3D features are calculated from an entire ROI volume. Thus, compared with 2D features, 3D features contain more tumor information but may also contain more interference due to, for example, noise and variations in slice thickness, and are less easy to obtain, slower to calculate, and more labor-intensive to generate (due to multiple-layer contouring being required) [60]. As a result, whether to use 2D or 3D image features remains a topic of debate. Wan et al. [61] evaluated the diagnostic performance of 2D and 3D radiomics features based on MR images of solitary pulmonary lesions (SPL). They found that the latter features performed better than the former features (AUCs: 0.824 vs. 0.740) and that combined features did not show better performance than either type of features alone (AUC = 0.813). Xu et al. [62] found that 3D radiomics features showed better predictive performance than 2D radiomics features in a study of multi-organ cancer, as unlike the latter, the former was significantly correlated with total lesion glycolysis, tumor volume, and staging [63]. However, Shen et al. [60] demonstrated that compared with 3D radiomics features, 2D radiomics features of CT images of non-small cell lung cancer (NSCLC) performed slightly better, and Zhu et al. [64] reached the same conclusion. Both studies have attributed these performance discrepancies between 2 and 3D radiomics features to the inconsistent resolution of CT images. However, there is no conclusive evidence that 2D features are superior to 3D features.

Researchers have also been searching for new quantitative imaging features to enrich radiomics investigations. Beichel et al. [65] evaluated the ability of 17 features of PET images, such as standardized added metabolic activity and rim average (RA; the mean of uptake in a two-voxel-wide rim region around an ROI), to predict the DFS of HNC patients. They found that RA may help differentiate between true- and false-positive recurrences of HNC. Buizza et al. [66] devised a new set of PET/CT image radiomics features (longitudinal patterns) to capture changes in the intensity at various distances from the border of a tumor. This set of radiomics features is superior to traditional radiomics features, as the latter are extracted from a defined ROI, meaning that useful information elsewhere in a tumor is often undetected. Moreover, there are two studies [44, 47] that developed a new form of radiomics features. That is, the researchers extracted features from images before and after treatment and then subtracted the post-treatment features from the pre-treatment features to obtain the radiomics features that were used to predict treatment response. These features can help to quantify peritumoral information that is complementary to intratumoral radiomics features. However, these quantitative imaging features are modality-dependent and have not been standardized, so they cannot yet be used as conventional radiomics features.

At present, radiomics studies typically extract large numbers of features from images using commercial or open-source software or software package, such as PyRadiomics [67], 3D Slicer [68], and Imaging Biomarker Explorer [69]. These radiomics features can be calculated by corresponding formulas. The Image Biomarker Standardization Initiative [70] was established to standardize the extraction of image features and thereby ensure the repeatability of feature extraction across different platforms.

#### Feature selection

Typically, hundreds of radiomics features are extracted as modeling candidates, and if all of these were used to construct a model, it would have excessive feature dimensions and be too complex, meaning that it would over-fit data and thus have low generalizability. Furthermore, most extracted radiomics features are highly correlated with each other, so reduction and feature selection must be performed before modeling. This is achieved using radiomics feature-selection methods, which select the most relevant features and remove the redundant features from a large number of features. Feature reproducibility should be considered during feature selection, as the aim of the latter is to obtain the optimal feature subset or feature representation that has the maximum correlation with the endpoints and the minimum correlation with other features [71].

**Fig. 3:**
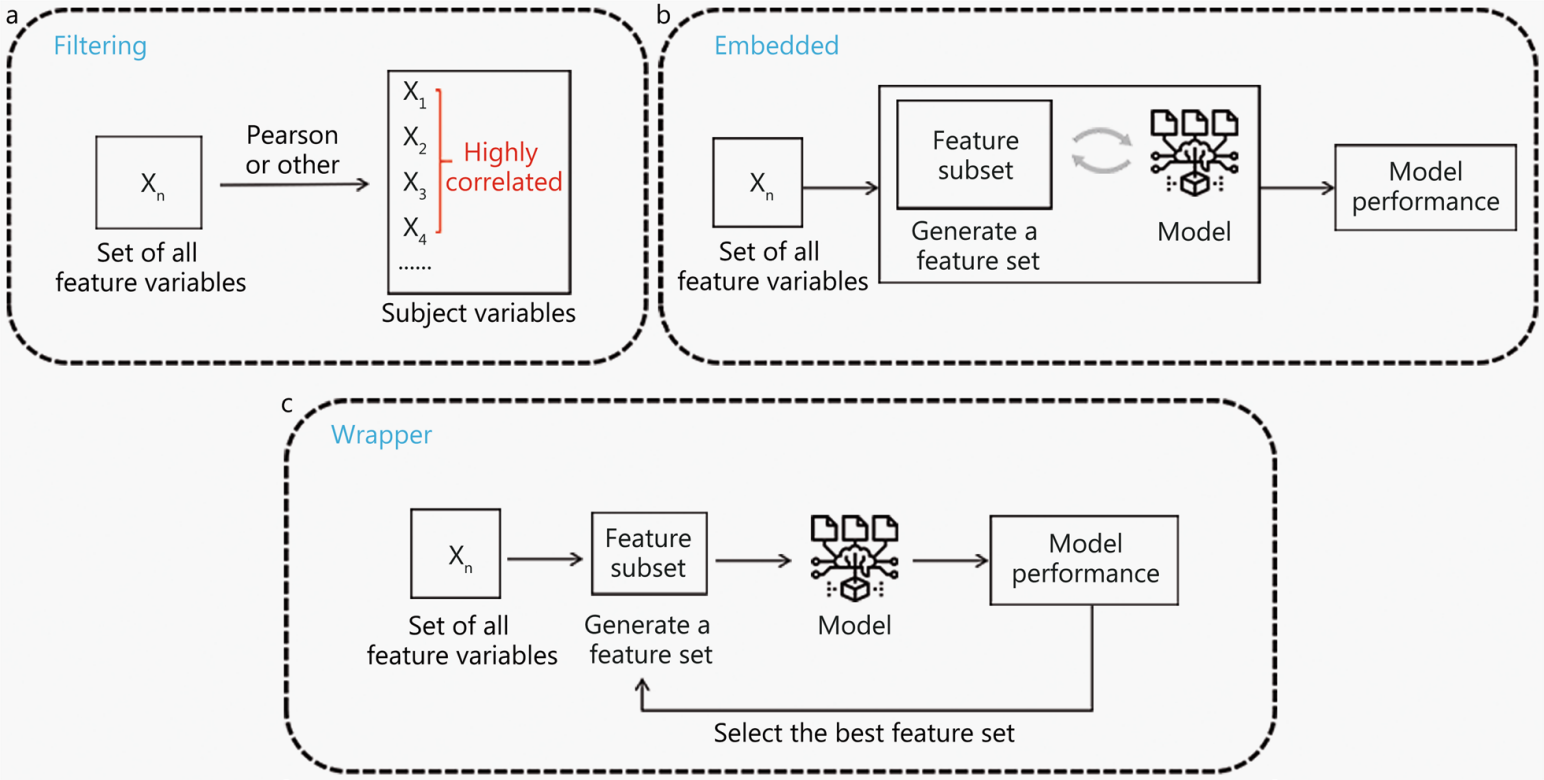
Feature selection. Feature selection methods including filtering, wrapper, and embedded. **a** The filtering methods rank the features according to a certain characteristic or correlation, and specify a threshold value or directly select the top ranked features. **b** The embedded method adopts the way that the feature is directly selected by the model. The model obtains the weight coefficient of each feature after trainings and selects the best feature according to the coefficient. **c** The wrapper methods take model performance as a criterion to judge the quality of features or feature subsets, and gradually retain or remove several features

Feature selection methods comprise filtering, embedded, and wrapper methods (Fig. 3). Filtering methods rank features according to the repeatability and their relevance to the endpoints. Then, the top ranked features or those that are above a specified threshold value are selected or excluded. Independent features are filtered by using the Pearson correlation method to exclude features with, for example, correlation coefficient > 0.75 (or some other pre-determined thresholds of correlation coefficient). Thus, in studies (e.g., [72]) that have used the Pearson correlation method to assess the correlation between tumor volume and radiomics feature values, highly volume-correlated features that meet a Pearson’s correlation threshold have been removed. Another filtering method is mRMR method [73], which aims to identify the best subset of features, maximize the relevance between subset and target variables, and minimize the redundancy between features based on mutual information. Hu et al. [74] used the mRMR method for dimensionality reduction in a radiomics study of nasopharyngeal carcinoma. Other filtering methods that have been used are Relief [75], Student’s *t*-test [76], and Chi-square test [77]. In addition, Parmar et al. [78] examined 14 filtering methods and found that features selected using the Wilcoxon test showed high stability (stability = 0.84 ± 0.05) in their training cohort. Wrapper methods employ model performance as a criterion to judge the quality of features or a feature subset; that is, they gradually retain or remove several features and finally select the feature subset that enables a given model to achieve optimal performance. For instance, recursive feature elimination (RFE) is widely used in radiomics: it generates a subset of features, iteratively constructs a model from the current feature subset, obtains the degree of importance of each feature, removes unimportant features, and retains the features with the best performance [79]. Yu et al. [80] adopted RFE for feature selection in their multiphasic CT-based radiomics analysis to differentiate benign and malignant parotid tumors, and used multiple methods for feature dimensionality reduction. In contrast to filtering methods and wrapper methods, embedded methods perform feature selection and model training simultaneously. First, a classifier obtains the weight coefficient of each feature after training, and then these coefficients are evaluated by a specific model to select the best feature, i.e., the feature is directly selected by the model. LASSO [81] is a commonly used embedded method that applies regularization to remove redundant features and retains the most relevant features. However, LASSO tends to ignore the pairwise correlations of features [82], so it must be combined with other feature redundancy elimination methods to enhance model reliability. In most radiomics studies, feature selection has been conducted via multiple steps using a combination of methods focused on different feature characteristics. For example, in a study of nasopharyngeal carcinoma [83], intraclass correlation coefficients (ICC) were first used to evaluate inter- and intra-observer agreement, and features with high reproducibility were selected. Then, the Wilcoxon rank sum test was used to select the radiomics features that statistically differed between regions of lymphatic infiltration and regions of non-lymphatic infiltration. Finally, LASSO was used to select the most relevant and independent features from a training set.

The feature selection methods described above are supervised methods. However, researchers have also employed unsupervised methods, such as principal component analysis (PCA) [84], and *t*-distributed stochastic neighbor embedding [85]. However, most radiomics studies have implemented supervised methods, so unsupervised methods are not reviewed here.

#### Feature reproducibility

**Fig. 4:**
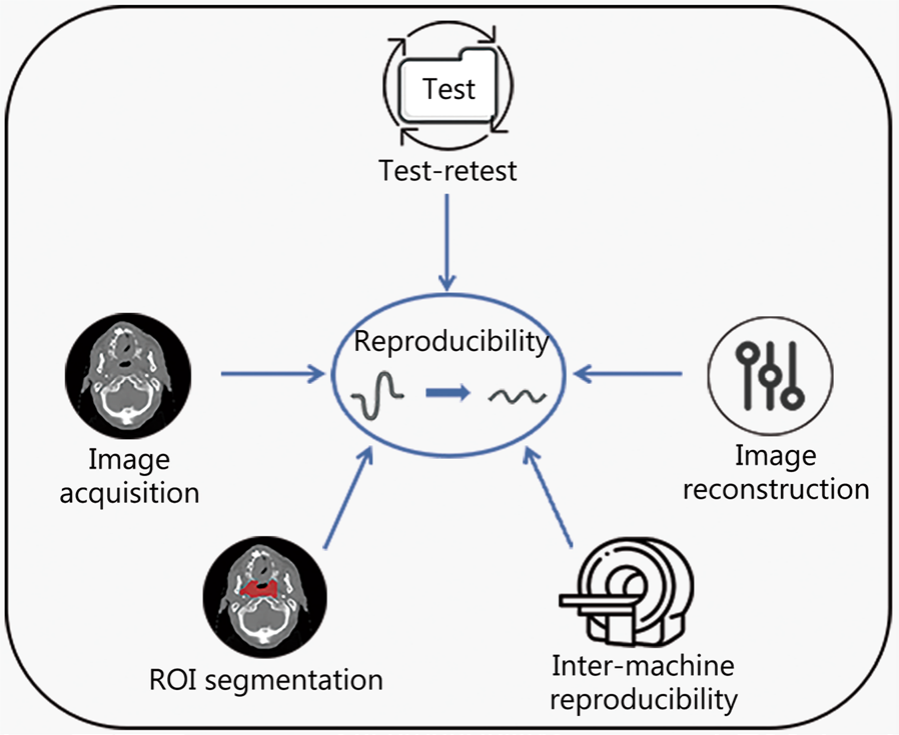
Influencing factors of feature reproducibility. The alteration of image acquisition details among the five influential factors can significantly affect the extracted features, resulting in varying outcomes. The stability of radiomics features is highly influenced by image reconstruction techniques. The commonly used filtered back-projection and iterative methods in radiomics research tend to decrease the stability of radiomics features. The test–retest strategy can be an effective tool in reducing the variability caused by image acquisition and reconstruction. The choice of ROI segmentation methods such as manual, semi-automatic, and automatic, and the size of ROI can contribute to different levels of feature reproducibility. Moreover, inter-machine reproducibility has a substantial influence on the degree of feature variation. ROI region of interest

Reproducibility is a measure of the variability of repeated measurements of the same or similar quantitative imaging biomarkers in a real clinical environment and is affected by external factors that cannot be strictly controlled, such as operators, measurement systems, and measurement equipment [86, 87]. Thus, reproducibility represents stability, so radiomics studies must ensure that the radiomics features they use have high reproducibility, such that their models generate similar classification results in different clinical environments. The values of features are affected by all of the steps prior to radiomics analysis, including image acquisition and preprocessing [88]. Therefore, during research, appropriate treatment should be performed as far as possible from the source of variation to obtain stable features with high reproducibility. Based on the radiomics workflow, we discuss the stability of features in terms of image acquisition, image preprocessing, characterization or segmentation of tumor areas of interest, and feature selection (as shown in Fig. 4).

Features are extremely sensitive to changes in acquisition details, even two images of the same tissue may yield different results due to differences in acquisition details [89]. This sensitivity usually affects the generalization performance of a final model. Balagurunathan et al. [90] conducted a test–retest study of lung CT images and found that the concordance correlation coefficient (CCC) ≥ 0.9 of radiomics features was only 30.14%. As it is unlikely that a tumor changes within a short period, these unstable radiomics features might have been due to the different postures of patients during rescanning. Midya et al. [91] found that image acquisition parameters (i.e., tube current and noise index) and reconstruction techniques strongly affected the reproducibility of CT-based radiomics features. There is inevitable noise interference in image acquisition, and Tu et al. [92] found that in the presence of the quantum noise inherent in CT images, the “ShortRunHighGrayLevelEmpha”, “ShortRunLowGrayLevelEmpha”, “LowGrayLevelRunEmpha” and “LongRunLowGrayLevelEmpha” features were the most stable, whereas the cluster shadow and maximum probability features were the most unstable. Image noise can also be reduced by increasing the tube current, as this increases the reproducibility of radiomics features [91].

Image reconstruction has a strong influence on the stability of radiomics features. For example, the filtered back-projection and iterative methods commonly used in radiomics research decrease the stability of radiomics features. Abundant noise is generated by filtered back-projection methods, but this can be removed (without changing noise texture) by reconstruction using deep-learning neural networks [93]. Yan et al. [94] explored the influence of reconstruction settings on textural parameters and found that they were influenced more by grid size than by the number of iterations or the full width at half maximum. Galavis et al. [95] determined that in different acquisition modes and using different reconstruction parameters, entropy-first order, energy, maximal correlation coefficient, and low-gray level run emphasis parameters exhibited small variations, which means that they have good reproducibility and can be considered good candidates for automatic tumor segmentation. Prayer et al. [96] explored the reproducibility of CT radiomics features of fibrosing interstitial lung disease (fILD) and found that slice thickness had a more significant impact than reconstruction kernels on the reproducibility of features between and within scanners. Compared with thin slices, thick slices are more appropriate for measuring tumor volume and volume changes [97], as thin slices increase noise levels, which can obscure texture features. However, thick slices reduce noise levels, but it also blurs the image.

Table 8 [95, 96, 98–102] lists studies that have investigated the reproducibility of radiomics features from the perspectives of scanner models or reconstructed environments and have focused on the identification of reproducible features. However, the reproducibility of these radiomics features cannot be directly compared or used.

**Table 8:** The summary table of literature focused on extracting reproducible features

Modality	Disease	Variability	Statistical indicators	Reproducibility summary of radiomics features	References
PET	Drenal gland carcinoma, Lung, Epiglottis, and esophagus cancer	Acquisition modes Reconstruction parameters	${\text{\% Diff}} = \frac{{100 \times \left( {{\text{X}} - {\text{X}}_{{{\text{mean}}}} } \right)}}{{{\text{X}}_{{{\text{mean}}}} }}$	Entropy-first order, energy, maximal correlation coefficient, low gray level run emphasis	[95]
CT	fILD	Scanners Reconstruction settings (reconstruction kernels, slice thicknesses)	ICC	Radiomics of fILD are highly repeatable for constant reconstruction parameters in a single scanner, intra- and inter-scanner reproducibility are severely impacted by alterations in slice thickness more than reconstruction kernel	[96]
CT	Lung, liver and kidney tumors	Segmentation variability	ICC	Reproducibility: shape features > first order features > GLCM	[98]
CT (Phantom)	Lung cancer	CT acquisition parameters Scanners	CCC, AUC	Tumor-mass, sigmoid-offset-mean, gabor-energy	[99]
CT	Liver tumor	CT radiation dose Reconstruction settings (reconstruction section thicknesses, reconstruction kernels, reconstruction algorithms)	Hierarchical clustering	Reproducibility: shape features (including the maximum axial diameter and volume) > other features	[100]
MRI	Cervical cancer	Scanners Segmentation readers	ICC	Reproducibility: shape features > other features	[101]
MRI (phantom)	Tumor	Scanners	ICC, COV	Reproducibility: first-order features > other features	[102]

*fILD* fibrosing interstitial lung disease, *ICC* intraclass correlation coefficients, *CCC* concordance correlation coefficient, *AUC* area under receiver operating characteristic curve, *COV* coefficient of variation, *GLCM* grey level co-occurrence matrix

Voxel-size resampling is a preprocessing step in image acquisition and reconstruction. It is important in CT, where voxel sizes affect a considerable proportion of radiomics features [103]. Voxel-size resampling can be accomplished using various interpolation algorithms, but these may use different resampling voxel sizes and box widths and thus may modify radiomics feature estimates in different ways [104]. Therefore, image interpolation should be performed at the same voxel size as much as possible. In addition, resampling may not be sufficient for some texture features. Thus, Shafiq-Ul-Hassan et al. [105] enhanced feature robustness through voxel size normalization, and Jensen et al. [106] corrected variability across different volumes of interest by converting CT images into parametric maps with a fixed voxel size. Bologna et al. [107] examined MR image radiomics and found that image preprocessing methods (Z-score normalization, resampling, Gaussian filtering, and bias field correction) significantly increased the robustness of radiomics features to different sources of variability (time of repetition and echo, voxel size, random noise, and intensity non-uniformity). However, Li et al. [108] found that image resampling, intensity normalization, and N4 bias field correction did not significantly affect the reproducibility of radiomics features, but the ComBat harmonization method removed most scanner effects and improved the reproducibility of features.

**Fig. 5:**
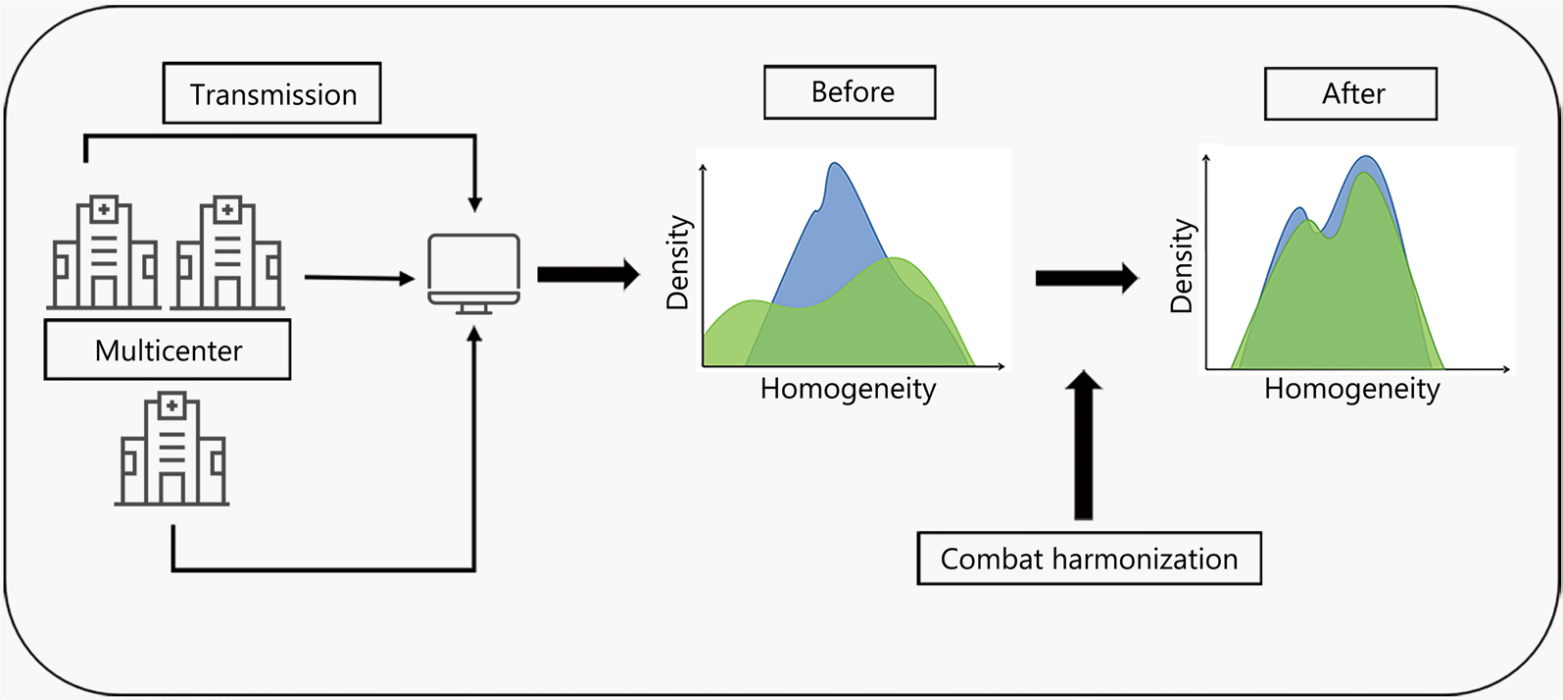
Workflow of ComBat harmonization. The multicenter data is obtained, and then the features in the images are extracted. The differences of the feature values are harmonized to obtain the normalized features

ComBat harmonization is a normalization technique that is widely used in radiomics, as radiomics features are easily affected by differences in acquisition equipment and reconstruction parameters, especially in multicenter studies. ComBat harmonization reduces these differences to lessen their impact on features, which enhances feature reproducibility [109]. For example, ComBat harmonization effectively eliminated the differences in MR radiomics feature values caused by heterogeneity of multicenter techniques, thus preventing reproducibility being affected [110]. As ComBat harmonization is a data-driven approach, it can be applied directly to extracted image features (i.e., without the need for retrieval of images), but it is recommended to be applied only after careful examination of the distribution of eigenvalues at the sites to be aggregated [111]. Crucially, ComBat harmonization eliminates the center effect while preserving some biological information associated with radiomics features [56, 112]. ComBat harmonization techniques have also been used in PET or CT radiomics studies. Figure 5 shows the basic workflow of ComBat harmonization.

Apart from applying ComBat harmonization, applying the same and appropriate reconstruction methods as much as possible and conducting test–retest studies are other ways to reduce variability in image acquisition and reconstruction. Manual segmentation remains the first choice in radiomics research; however, it may be replaced gradually by automatic and semi-automatic segmentation, given the continuous improvements in the Acc of the latter methods. Compared with manual segmentation, automatic segmentation reduces inter-observer variability, leads to better reproducibility and robustness, and is faster [113, 114]. The reproducibility of manual segmentation can be increased by using multiple experts to perform segmentation. Usually, segmentation was performed by two or three experienced experts and then evaluated by comparing ICC. In addition, contouring protocols or guidelines can be used to reduce inter-expert variation in target volume delineation [115]. Gitto et al. [116] used a time-saving method based on geometric transformations of an ROI, which can simulate multiple manual delineations, to evaluate PET/CT radiomics feature stability. They found that over 76% of radiomics features were stable to ROI transitions. However, Jensen et al. [88] emphasized that ROI size must be considered in radiomics, as MR-derived features are more robust than CT-derived features to changes in ROI size. Denzler et al. [117] found by examining phantoms that a larger ROI corresponded to a higher percentage of intensity feature stability and suggested that non-contrast-enhanced CT lung images exhibit tissue- and disease-specific radiomics feature stabilities.

New feature-selection methods have also been developed to improve stability. Bologna et al. [118] developed a method that can be used to assess feature stability and perform preliminary feature selection based on a single acquisition and an ROI. Yan et al. [119] devised a novel method, named stability selection supervised PCA, that identifies stable features from radiomics big data and applies dimension reduction to achieve right-censored survival outcomes. Lam et al. [120] constructed a selection algorithm that determines optimal feature selection combinations. This algorithm also ensures selected features to have good AUCs and stability scores. Feature output stability is assessed on the basis of 10 iterations, and the stability scores are quantified by a frequency-based criterion. The retained test dataset for each iteration is used to evaluate the AUC. The product of the two scores for each feature selection combination is determined, and the combination with the highest score product represents the optimal feature selection combination. Flouris et al. [89] developed a CT simulator that reconstructs images under different noise levels using different reconstruction algorithms, which may have novel applications in automatic processing of multicenter datasets.

Compared with features with low reproducibility, features with high reproducibility exhibit greater resilience to environmental changes. Radiomics studies usually quantify the reproducibility or stability of features using several types of indicators, such as ICC, coefficient of variation (COV), or CCC. These indicators are commonly used to measure the inter-observer stability of radiomics features but may also be used to perform dimension reduction for feature selection (as mentioned in the subsection: Feature engineering). ICC and CCC are the most commonly used indicators, for which threshold values are typically assigned to allow the relative stability of features to be determined. However, there is no consensus on the threshold values for these two indicators, and ICC values obtained from a test–retest analysis cannot be directly compared with those obtained from an inter-observer analysis [121]. Furthermore, the COV index is often used to provide information on the variability of a feature measurement unit [121].

#### Feature interpretability

Radiomics mining is entirely data-driven and interprets imaging data quantitatively rather than qualitatively, meaning that it can obtain much information that is difficult to obtain visually. Radiomics features can also be well defined at a mathematical level, which endows them with a certain level of interpretability. However, there is a lack of interpretability of radiomics features at the biological level, which may limit the application and development of radiomics in medicine. In recent years, there has been an increase in radiomics studies based on deep learning, and deep learning-based radiomics models can outperform conventional radiomics models [122, 123]. However, deep learning-based radiomics is a “black box”, as deep features do not have accurate formulations and definitions and thus cannot be conceptualized. Moreover, although deep features can be explained at the feature level by methods that link them to traditional radiomics features and semantic features [124], the interpretability of deep features remains low. In this part, we focus on the interpretability of traditional radiomics features.

**Fig. 6:**
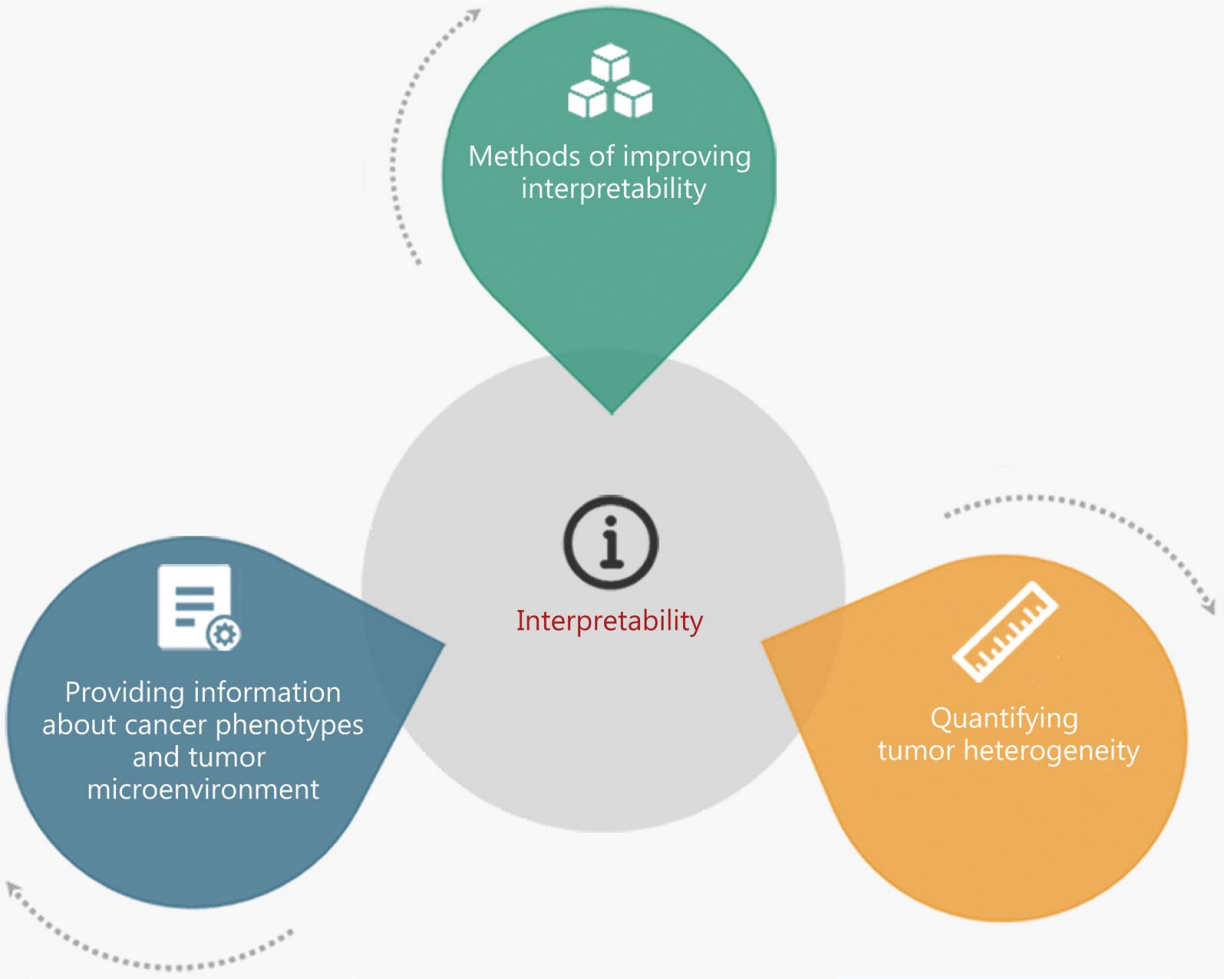
Three pathways of improving feature interpretability. The interpretability of radiomics features at the biological level can be enhanced by providing information about cancer phenotypes and tumor microenvironment, quantifying tumor heterogeneity, and a number of methods of improving interpretability

Although some studies (such as those described in the clinical applications based on radiomics section) have demonstrated the predictive and diagnostic power of radiomics in applications related to cancer, the interpretability of radiomics features is limited and does not meet the needs of clinical experts. Therefore, associated biological backgrounds must be supplied with radiomics features to increase their interpretability. As shown in Fig. 6, this is generally achieved via three approaches: by determining the biological significance of features, by quantifying tumor heterogeneity, and by developing methods to improve feature interpretability.

Radiomics features provide information about a cancer’s phenotype and a tumor’s microenvironment that is distinct from and complementary to other data, such as clinical and genomic data [8]. Aerts et al. [125] used gene set enrichment analysis to compare radiomics features with gene expression profiles and found that the features were significantly correlated with different biologic gene sets. Moreover, Rifi et al. [126] found that there were significant differences in the radiomics features between different cell lines, suggesting that features can be grouped according to their behaviors. As radiomics features themselves contain information relevant to a given research objective, and this information may be related to gene expression, identification of this information can offer biological perspectives that are not offered by traditional imaging.

Identification of tumor heterogeneity during tumor treatment can help evaluate the effectiveness of treatment and thus guide treatment planning, and also reveals the individuation of a patient’s tumor. Therefore, exploring the association between tumor heterogeneity and texture features can enhance the interpretability of radiomics features. Some first-order histogram features such as kurtosis, skewness, percentiles, and their respective changes are reliable quantitative proxies of tumor heterogeneity and more directly correlated than other features with potential physiological structural changes that occur during the progression of both treated and untreated tumors [127]. However, one disadvantage of histogram-based estimation of tumor heterogeneity is that it ignores the spatial structure of a tumor [128]. Wang et al. [129] divided patients into groups according to their radiomics scores and found that the tumor immunity and tumor microenvironment of the high- and low-scoring groups were different, indicating that radiomics could reflect the heterogeneity of tumors. Similarly, other researchers found that CT features based on the fourier transform are potentially useful for quantifying tumor heterogeneity in lung cancer patients and that radiomics features associated with tumor heterogeneity are correlated with OS [130]. Moreover, texture features describe the distribution pattern of voxels and can be used to quantify intra-tumor heterogeneity [131]. Thus, radiomics features can be employed to quantify tumor heterogeneity, and the correlation features of histograms can enhance understanding of tumor heterogeneity.

Several methods have been developed to improve the interpretability of radiomics analyses. Vuong et al. [132] devised a method for creating radiomics feature-activation maps that allows identification of spatial-anatomical locations responsible for signature activation based on local radiomics. Kuthuru et al. [133] adopted a dictionary learning approach to derive visually interpretable imaging features. In addition, Luo et al. [134] proposed an approach which enables exploration of hierarchical relationships between biophysical features based on a Bayesian network.

In addition to the above-described three approaches, the literature suggests another approach that can be used to improve the interpretability of radiomics features from a biological or clinical-physiological perspective. This approach is based on the fact that the semantic features of clinical reports may be more explanatory than traditional radiomics features. Therefore, quantification of explanatory semantic features and integration of the resulting quantities into a radiomics framework may improve the overall interpretability of radiomics from a clinical-physiological perspective. For example, in a 2021 paper by Choi et al. [135], an interpretable spiculation feature based on spiculation quantification was used for radiomics modeling. The model achieved an AUC of 0.82 on the Lung Image Database Consortium dataset and an AUC of 0.76 on a LUNGx dataset. In 2022, Choi et al. [136] released a large-scale dataset, the Clinically-Interpretable Radiomics Dataset, which focuses on features on the surface of pulmonary nodules such as spiculation or lobulation and sharp or curved spikes, as these features can be clinically explained. For example, the formation of spiculation can be explained by the proliferation of fibrous connective tissue caused by the infiltration of or stimulation of surrounding tumor cells.

### Statistical modeling

After feature engineering, a suitable model is developed based on the selected features. Researchers can choose a single machine learning algorithm to generate a model and then evaluate its performance, or use several algorithms to generate various models and then compare their performance to identify the best model. It is currently believed that no algorithm is the best in all scenarios, so researchers must choose the appropriate algorithm for a given scenario, which may be an SVM [137], a logistic regression [138], a KNN [139], a decision tree (DT) [140], a RF [141], or an extreme gradient boosting (XGBoost) algorithm [142]. In recent years, due to advancements in deep learning technology, researchers have increasingly used deep learning methods to construct models.

We summarize the strengths and limitations of some algorithms and classify them into machine learning methods, deep learning methods and statistical methods. In the second part of this paper, we extract various algorithms from the references. Table 9 focuses on some common algorithms in these references. Statistical methods are often used to evaluate the data differences between training sets and validation sets, and the differences between survey groups. In addition, Kaplan–Meier analysis and log-rank test are generally combined to compare the OS of different risk groups, so these two methods generally appear simultaneously in prognosis research. The application of machine learning methods is diverse, including feature selection, model construction, model performance validation and so on. About feature selection, it has been described in detail in the subsection: Feature selection of feature engineering. Some algorithms are both common in feature selection and model construction, such as LASSO and SVM. Traditional machine learning methods usually divide the problem to be solved into several sub-problems and then solve them one by one. Deep learning methods solve problems in an end-to-end way, which means the feature engineering step is not required. However, correspondingly, deep learning methods are less interpretive and have higher requirements for machines and equipment.

**Table 9:** Strengths and limitations of commonly-used models

Type	Method	Strengths	Limitations
ML	PCA	It remains most of the main information and has simple calculation process	It would lose some important information and the Interpretation is poor
	mRMR	It is suitable for handling multiple classification tasks	The correlation between feature crosses and target variable is ignored
	LASSO	It is a good solution for solving multicollinearity problems, and the results are easy to interpret	It tends to select one of a set of highly correlated features
	CV	It can evaluate the model more reasonably and accurately and obtain more useful information from limited data	The computation is increased
	SMOTE	The overfitting problem of simple over-sampling is overcome	It requires repeated adjustment of important parameters
	LR	It has low computation cost, fast computation speed, and is easy to understand and implement	It only handles binary classification tasks and is easy to underfit
	SVM	It can solve high-dimensional problems and has strong generalization ability	It can only handle binary classification tasks (conventional SVM) and the efficiency of training large sample is low
	KNN	It is suitable for nonlinear classification, and has high Acc	It requires a lot of memory, and when the sample is imbalanced, the deviation of prediction is large
	DT	It can be analyzed visually, and the running speed is fast	It is easy to overfit and overlook the correlation of attributes in a dataset
	RF	It is suitable for handling high dimensional data, and the ability to adapt to datasets is strong	It is not good at dealing with low dimensional data, and it is much slower than DT
	Cox regression model	It has great flexibility and no requirement on data distribution	The best fitting effect for each data may not be achieved
	Naïve Bayes	It is easy to understand the interpretation of the results, and performs well on small datasets	It is sensitive to the form of input data
DL	3D-CNN	It is easy to handle high-dimensional data, and the feature extraction process is automatic	It is difficult to interpret results and lots of valuable information may be lost
	ANN	It has high classification Acc and strong robustness and fault tolerance	It is difficult to interpret results and requires a lot of parameters
SM	*t*-test	It is easy to explain, has strong robustness and can control individual difference well	It can not be used for multiple comparisons, only to compare whether the difference between the two averages is significant
	Mann–Whitney *U* test	There is no requirement for data distribution	When the data conforms to normal distribution and the variance is homogeneous, the test efficiency is lower than the *t*-test efficiency
	Spearman correlation analysis	It is suitable for nonlinear relations and continuous and discrete datasets	It is less efficient than Pearson correlation coefficient
	Kaplan–Meier analysis	It provides a variety of test methods, and is easy to implement	It can only perform univariate analysis
	Log-rank test	It analyzes the data in combination with all time points	It requires meeting equal proportional risk assumptions and only performs univariate analysis
	Fisher’s exact test	It is suitable for small samples and can accurately calculate the significance of deviations from the null hypothesis	It can only applicable to sample size *n* < 40 or theoretical frequency T < 1
	Chi-square test	It is convenient, concise, and widely used	It is more complex than *t*-test and the test efficiency is lower than *t*-test efficiency

*ML* machine learning, *SM* statistical method, *DL* deep learning, *PCA* principal component analysis, *mRMR* maximum relevance minimum redundancy, *LASSO* least absolute shrinkage and selection operator, *CV* cross validation, *SMOTE* synthetic minority over-sampling technique, *LR* logistic regression, *SVM* support vector machine, *KNN* K-nearest neighbors, *DT* decision tree, *RF* random forest, *CNN* convolutional neural network, *ANN* artificial neural network, *Acc* accuracy

Researchers typically use the following metrics or methods to evaluate model performance: AUC, Acc, F-1 score, sensitivity, specificity, precision, positive and negative predictive values, calibration curve analysis, decision curve analysis, the Hosmer–Lemeshow test, or the Akaike information criterion. These reveal certain characteristics of a model, such as its discriminability, generalizability, robustness, clinical utility, and goodness of fit. Nevertheless, many aspects remain to be improved in radiomics models. Here, we review four of these aspects.

#### Model generalizability

The generalizability of a model is its ability to predict unknown data. The smaller the deviation of a model-predicted result from the real result, the higher the generalizability of the model. Thus, a model with high generalizability is better than a model with low generalizability. Accordingly, generalizability must be considered when radiomics models are applied in clinical decision making.

There are many reasons why a radiomics model may exhibit low generalizability. The root cause may be a difference between the distribution of the training data and that of the unknown data. For example, as there is currently no standard workflow for radiomics, changes in any aspect of a workflow, such as data sources, scanners, acquisition protocols, or image segmentation methods, will cause some level of data discrepancy that will affect the performance of a model. Castillo et al. [143] found that radiomics models had an excellent ability to distinguish between low- and high-grade prostate tumors when single-center data or single-scanner data were used, but this ability was significantly reduced when multicenter data and/or multi-scanner data were used. Therefore, standardizing these processes can reduce variability and improve generalizability. However, as it will take some years for this to be achieved, another approach must currently be used: examining the number and diversity of input samples. Therefore, researchers have attempted to develop models using multicenter data and to circumvent the ethical and legal problems raised by multicenter data sharing by using distributed learning, which aims to train radiomics models without requiring the data to “leave” the hospital where it is housed. For example, Bogowicz et al. [144] constructed centralized and distributed HNC survival-prediction radiomics models and found that these models exhibit no significant AUC differences in terms of feature selection and classification. This confirmed that distributed learning does not affect model performance and indicates that it is a valid new approach for adopting multicenter data to generate models with good generalizability.

When a large amount of data cannot be obtained, generalizability can be improved by small sample learning and manifold learning. Small sample learning incorporates data augmentation and transfer learning [145]: data augmentation increases sample size and diversity [146], while transfer learning allows knowledge to be learned from related domains to increase model performance in the target domain. A study published in 2022 [147] described a feature extraction method based on transfer learning that increases the number of samples, suggested that the deep radiomics features extracted by this method might be more accurate than manually extracted radiomics features, and then confirmed this by comparing the performance of models for predicting the regression of early HNC. Transfer learning involves domain adaptation: the adjustment of a model to enable it to adapt to different domains (i.e., different datasets), so the model exhibits good generalizability (i.e., can be applied to unfamiliar sample sets) [148]. For example, Wang et al. [149] performed cross-phase adversarial domain adaptation using a gastric CT arterial phase as the source domain and the pre-contrast phase as the target domain, and thereby obtained a radiomics model that performed well in predicting the target domain. In addition, manifold learning can increase the number of samples by reducing the dimensionality of high-dimensional image data. Accordingly, Zhang et al. [150] developed a classifier called multi-kernel regression with graph embedding, which applies manifold learning to prevent a radiomics model overfitting when predicting distant metastases of nasopharyngeal carcinoma. The classifier embeds a class compactness graph and connects a pair of samples of the same class in low-dimensional label space via undirected weighted edges to understand their relationship. Then, it maximizes the closeness between samples of the same class to prevent overfitting and enhance generalizability.

Researchers have also improved models’ generalizability by employing appropriate feature-selection approaches. Shayesteh et al. [151] used the mutual information feature-selection method to enhance the generalizability of radiomics models; specifically, they analyzed non-linear relationships and linear relationships to find radiomics features with the highest discriminability, which they used to enhance model performance. As a target dataset may be nearly identical or completely different from an original dataset, the repeatability and reproducibility of features are the factors influencing a model’s generalizability. Therefore, enhancing the reproducibility and reproducibility of radiomics features can also enhance a model’s generalizability. As such, the harmonization schemes are crucial, such as ComBat. For example, a study published in 2022 [152] demonstrated that specific post-processing algorithms can be applied to coordinate PET image noise and thereby increase the agreement of radiomics features. Another study [153] developed convolutional neural networks (CNNs) to coordinate CT images with different reconstruction kernels, which helps to improve a feature’s reproducibility and thus a model’s generalizability. Moreover, data augmentation is an image coordination method. Ibrahim et al. [154] adopted the radiomics feature harmonization scheme of ComBat harmonization and found that it improved feature agreement when the acquisition and reconstruction parameters were significantly different. However, they also noted that direct application of the ComBat method was not invariably successful, such that pre-adjustment of the parameters of the phantom dataset was necessary. Image coordination has also been achieved by identifying robust features. Robinson et al. [155] found that a model’s generalizability decreased monotonously with a reduction in feature reproducibility, meaning that the classification generalizability of the model was improved by selecting radiomics features with high robustness.

Several studies have been performed to select features with high repeatability and reproducibility for use in feature engineering. Lu et al. [99] developed a new phantom-based framework to screen radiomics features for repeatability and reproducibility and identify robust features by evaluating the effects of biological and noise signals. A study [156] published in 2021 used a new method (which differs from embedded methods) for selecting robust features for predicting the mutation status of isocitrate dehydrogenase 1/2 (IDH1/2) in glioma. In this method, feature selection involves the identification of variables that are part of causal structures (based on causal reasoning), are insensitive to environmental changes, are highly robust, and have a constant relationship with the IDH1/2 mutation status. Radiomics models were constructed based on these variables, and their generalizability and performance could be improved even in a set of non-observational environments.

#### Imbalanced data classification

In an imbalanced data set, there are large differences between the sizes of different classes of data. The generalizability of a radiomics model based on such a data set will be affected by these differences, which may decrease its predictive power. That is, in a case of a class imbalance, a model first learns that there is more of one class of data than another in the training set, so the model’s predictions are biased toward the majority class [157]. For example, if there are two classes of data, class 1 and class 2, and they are present in a ratio of 1:100, then the predictive Acc of a model based on class 2 data may be much higher than class 1 data. However, if we mainly focus on the 1 class, the model will not meet our requirements.

Resampling techniques are essential for dealing with class imbalances and have been used in many radiomics studies. These techniques increase the balance of an imbalanced dataset at the data level and involve either over-sampling or under-sampling. The former is performed by copying the minority class data to add more data [158], while the latter is performed by discarding a large amount of the majority class data. Regarding under-sampling techniques, a radiomics study mentioned above [149] developed the so-called sensibly based under-sampling imbalanced integrated framework, which divides the majority class data into different blocks by clustering data on the basis of sample sensitivity. This under-sampling technique comprises two steps. In the first step, a coordinated method is used to maintain the same sensitivity level in each block. In the second step, a self-paced factor is applied to reduce the sample probability of the block with a large sample size and is combined with multi-kernel regression with graph embedding to train a good radiomics model. Regarding over-sampling techniques, a study that categorized pathologic complete responses (pCRs) for cancer [159] included 222 patients (61 pCR and 161 non-pCR patients) in its training set. The two types of patient samples were significantly different, so the researchers applied the synthetic minority over-sampling technique (SMOTE) algorithm to an MRI-based radiomics feature set to balance the minority and majority differences between the samples. Although SMOTE is an over-sampling technology, it synthesizes similar new samples from original samples rather than copying original samples. In addition, Zhang et al. [160] developed a novel over-sampling network—DeepSMOTE, and pioneered the integration of convolutional image features with radiomics features to effectively enhance the classification ability of an unbalanced dataset. The target data for this study were rare rim-positive lesions present in quantitative susceptibility mapping. DeepSMOTE increases these data by adding the two nearest neighbors of each rim-positive lesion, and then linearly combines the features of each lesion and its nearest-neighbor to generate comprehensive data.

Researchers have also adopted algorithm-based techniques to address data set class-imbalance problems and thus alleviate the degradation of radiomics model performance. Ensemble learning, for example, combines several weak algorithms to obtain a more comprehensive and strong algorithm that performs bagging, boosting, and stacking, and studies have confirmed that ensemble learning-generated algorithms can be well applied to imbalanced data [161, 162]. In 2022, Tang et al. [163] reported the advantages of bagging ensemble learning for prognostic prediction of HNSCC and showed that a bagging ensemble radiomics model generated more accurate predictions (an average Acc of up to 88.3%) than DT, RF, XGBoost, SVM, and linear models. Another study [164] compared the predictive performance of four DT and ensemble radiomics models based on boosting with that of an SVM model and found that the performance of the former models was superior to that of the latter model. Cost-sensitivity learning is also an effective approach that can yield excellent results by increasing the cost of algorithmic misclassification [165]. In 2021, Sun et al. [166] combined cost-sensitivity learning with ensemble learning, as this is effective for application to imbalanced datasets. Moreover, a tiny class can be detected and then treated as an anomaly. For example, Welch et al. [167] used isolation forest algorithms to detect abnormal data and solve a data set class-imbalance problem when applying two pipelines (i.e., machine learning and deep learning pipelines) and synthesizing patient-specific features, clinical features, radiomics features, and quantitative radiation therapy features to predict the local failure of HNC treatment.

Furthermore, researchers have combined data-level approaches with algorithm-based techniques. Jiao et al. [168] extracted the radiomics features of US images and then constructed a cost-sensitive SVM model, a SMOTE and adaptive boosting (AdaBoost) combination model, and a random under-sampling and AdaBoost combination model (RUSBoost), for the original unbalanced data set. They also built a SVM model and an AdaBoost model and applied them to the original unbalanced data set and an adaptive synthetic-nominal (ADASYN) algorithm-balanced data set, respectively. All of the models obtained good results, with the RUSBoost model exhibiting the best performance.

#### Multi-modality fusion

The use of multi-modalities is an unsolved challenge in many fields, including in radiomics, where researchers must decide what modes to incorporate into studies and when to do so [169]. Many radiomics studies have adopted multi-modality fusion approaches and have concluded that a radiomics model based on multi-modal information is superior to a radiomics model based on single-modality information. Generally, there are three time nodes for multi-modality fusion, namely early fusion, intermediate fusion and late fusion [170], and the advantages of each are described in the “Limitations and suggestions” section.

Early fusion is also known as data-level fusion and is the fusion of multiple modalities’ information before a feature input classifier is implemented [171]. Li et al. [172] combined the MR image features of different sequences to construct radiomics signatures and then combined these with clinic-radiological risk factors to develop a multi-factor model based on a training set. They found that the multi-factor model had the highest performance. Another study [173] modeled radiomics features with the tumor–node–metastasis stage primary tumor volume, clinical and biological features, respectively, and found that the performance of the multi-factor model was better than that of the single-factor model. Sheikh et al. [174] compared a CT model, an MRI model, and a CT–MRI hybrid model, and found that the hybrid model achieved the highest AUC in predicting acute radiation-induced xerostomia in HNC, as their respective AUCs were 0.57, 0.66 and 0.70 in the external validation cohort.

Intermediate fusion is also known as inter-layer fusion as it is the fusion of modalities between the input and output layer during modeling [175]. A study published in 2021 [120] explored the ability of multi-omics models to predict the eligibility of patients with nasopharyngeal carcinoma for adaptive radiation therapy by using multi-kernel learning algorithms to achieve intermediate fusion of four types of omics features: radiomics, dosiomics, contouromics, and morphology features. Specifically, four single-omics models (a radiomics model, a dosiomics model, a contouromics model, and a morphology model) and four multi-omics models (a radiomics–dosiomics model, a radiomics–contouromics model, a radiomics–morphology model, and a radiomics–morphology–dosiomics–contouromics model) were constructed and studied, and decision graphs were used to select which model was the best, i.e., which combination of the above-mentioned omics features formed the highest-performing model. The results revealed the superiority of the radiomics features: the AUC of the radiomics model (0.94) was the highest of the four single-omics models, and radiomics features comprised the largest proportion of features in the multi-omics models. These results also indicated that the performance of multi-omics models was generally better than that of single-omics models.

Late fusion is also known as decision-level fusion as it involves building a model by performing a certain processing fusion of different modalities to enhance the model’s performance. Chen et al. [176] adopted a late fusion approach to devise a mixed predictive model. That is, they established a novel many-objective radiomics model and a 3D-CNN model, and then applied an evidence-reasoning method to fuse the outputs of these two models to obtain a higher predictive Acc than that obtained from either model alone. They also fused two kinds of image data: PET data and CT data. Their results revealed that the mixed model input with PET and CT data exhibited a predictive Acc superior to that of a single model input with only CT or PET data.

Other multi-modality fusions can be employed, such as radiomics feature and gene signature fusion or radiomics features fusion based on PET and MRI data. Researchers have yet to fully explore all forms of multi-modality fusions, so this remains a fruitful and exciting avenue of enquiry.

#### Model interpretability

The “black box” characteristics of the machine learning approaches used to construct radiomics models, combined with the high sensitivity of radiomics features to image-specific variations [177], mean that radiomics models can be complex. Moreover, most radiomics studies have been single-center retrospective studies and thus had inherent defects such as small sample sizes and ineffectively verified model generalizability. Furthermore, the decision-making processes of deep learning models are opaque and may be unknowable. All of the above-mentioned aspects mean that radiomics models may have low interpretability, i.e., the results of models cannot be explained and so are not trusted by doctors. In these cases, the clinical implementation and development of models are severely hindered.

There is a growing body of research focusing on solving interpretability problems. Thus far, most of this research has explored either the local interpretation of specific predictions or global interpretation of working principles [178]. In machine learning, linear models and DT algorithms are inherently globally interpretable. In contrast, the local interpretable model-agnostic explanations (LIME) technique makes use of prediction samples and perturbed samples generated by random perturbation to fit a simple interpretable model, which provides local explanations for a black-box model. Zafar et al. [179] constructed a deterministic LIME framework that uses hierarchical clustering and a KNN algorithm instead of random perturbations to enhance the stability of explanations. In addition, shapley additive explanations (SHAP) and LIME have often been compared, and SHAP has been a common method of interpretation. For example, Giraud et al. [180] used SHAP when developing a radiomics model for oropharyngeal cancer, determined SHAP values to quantify the contribution of each feature to predicting local recurrence, and used an interpretable method to identify the most important risk factors.

Partial dependence plots (PDPs) are applied for the global interpretation of working principles as they express the relationship between prediction targets and variables (features), which renders a black box model visible and thereby effectively increases interpretability [181]. Accumulated local effects (ALE) plots are a superior alternative to PDP, and an ALE plot was used by Tan et al. [182] to reveal the major effects of each radiomics feature they examined. Their ALE plot demonstrated that higher “GreyLevelNonUniformity” values reflected intratumoral heterogeneity, while lower “Strength” values and more image-defined risk factors were associated with a higher probability of MYCN oncogene amplification. Permutation importance is also a key technique, whereby features are constantly adjusted during the testing of a model and the importance of all of the features to the predictive ability of the model is evaluated by observing changes in the performance of the model [183]. Enke et al. [184] applied permutation importance to screen the most relevant predictive radiomics features from 100 ranked radiomics features. They concluded that the features on the Laplacian of a Gaussian-filtered image were the most important when all of the features were included, while the shape features were the most important when only the radiomics features of the original image were included.

One study [185] simplified selected radiomics features to enhance the interpretability of a model. It used only 5 of 42 related features and the whole omental tumor volume to build a model; this involved discarding most of the uninterpretable features, thus making the model easier to interpret than a model that included all of the features. Moreover, this simplified model maintained a high predictive power (AUC: 0.68 ± 0.03) on the external test set. Another study [186] used a gene masking technique to improve physicians’ acceptance of a model. Specifically, the gene expression profiles of patients were obtained, and the expression of related genes was retained while that of unrelated genes was masked. Then, the masked expression data was input into the radiomics model, and the predictive performance of the model was calculated. This process was repeated for the entire cohort, with a higher performance indicating a stronger predictive correlation between the gene set and the radiomics features. The above mentioned study in 2020 [132] also increased interpretability to produce a radiomics feature-activation map, which revealed that peritumoral regions were more relevant than GTVs for distinguishing histological subtypes of NSCLC in CT imaging.

The interpretability of black-box models can be improved via many other methods, but these have yet to be applied in radiomics. We hope that future research will increase the diversity of methods available to solve the interpretability problems of radiomics models.

## Discussion

### Summary and analysis

In this review, we review research on the application of radiomics for clinical diagnosis, prognosis, and determination of treatment responses, as well as research examining the two most important steps in radiomics analysis: feature engineering and statistical modeling.

#### Feature engineering

Feature engineering consists of feature extraction and feature selection. Feature extraction can be performed to extract 2D or 3D image features, and there is no consensus on whether either type of features is superior. Each type has its own advantages and can be selected according to the imaging method used or the nature of the research being performed. In addition, researchers have attempted to identify new radiomics signatures. Feature selection is conducted to reduce the redundancy between features and improve the correlation between features and clinical goals. Feature selection methods comprise filter methods, embedding methods, and wrapper methods. The latter methods exhibit overfitting problems and thus have rarely been used in radiomics research, and none of the remaining methods are suitable for all situations. The reproducibility of radiomics features is a key concern in radiomics research. Reproducibility is sensitive to external factors, and all of the steps preceding radiomics modeling can affect feature reproducibility. The reproducibility of features can be investigated by following the radiomics workflow: image acquisition, image preprocessing, delineation of tumor ROIs, and feature selection. Features are extremely sensitive to the changes in acquisition details; even two images of the same tissue site may be different due to differences between their acquisition details. Nevertheless, in various acquisition modes and with certain reconstruction parameters, some features exhibit good reproducibility and therefore are good candidates for tumor segmentation. Inter- and intra-scanner feature reproducibility is affected by the slice thickness during image acquisition: thick slices are best for measuring tumor volumes and changes in these volumes, as thin slices contain comparatively more noise, which obscures texture features. Various methods are used for image preprocessing; a common method is resampling of voxel size, which can solve the problem of different voxel sizes to enhance the reproducibility of radiomics features. In addition, the ComBat harmonization method can eliminate most scanner effects and improve feature reproducibility and thus is suitable for multicenter research. Although manual segmentation is the preferred method for delineating tumor ROIs, it may be replaced gradually by fully automatic and semi-automatic segmentation due to the continual improvements in the Acc of the latter two methods. The reproducibility of manual segmentation can be improved by its being performed by several experts, followed by inter-expert evaluation. Contouring protocols or related guidelines are recommended for reducing inter-expert variability in target volume delineation. Some studies have devised new feature selection methods to obtain radiomics features with high reproducibility. Radiomics features can be clearly defined at a mathematical level, which adds some level of interpretability to radiomics results, but radiomics features’ lack of interpretability at the biological level limits the application and development of radiomics in medicine.

#### Statistical modeling

The generalizability of models has received additional attention, as it determines whether models can be applied in real-world multicenter scenarios. The root cause of generalization problems is the differences between the distribution of training samples and that of testing samples, which originate at every step of the radiomics workflow. Therefore, a standardized radiomics research plan must be determined in future research. At this stage, this problem can be solved by using techniques such as transfer learning and manifold learning. In addition, incorporating robust features into a model can effectively improve its generalizability because, compared with features with low robustness, features with high robustness are better able to resist environmental changes. Imbalanced datasets are another factor that adversely affects a model’s generalizability and predictive ability. This problem has often been solved by employing data-level resampling techniques, such as over-sampling and under-sampling. In addition, an algorithm-level imbalance processing strategy has exhibited some advantages, such as its ability to construct a more sensitive loss function and a more reasonable integration strategy than would have otherwise been available.

Furthermore, multi-modal fusion and model interpretability have also been the focus of some attention in radiomics modeling. Research has shown that multi-modal fusion methods encompass one of three time nodes: early fusion, intermediate fusion, and late fusion. Regardless of the fusion method used, studies have revealed that compared with single-modal fusion, multi-modal fusion can obtain more mode information and thereby afford better prediction results. For example, a multivariate model that combines radiomics features with clinical factors was shown to have better predictive performance than a radiomics features-only model or a clinical factors-only model. Analogously, the performance of fusion models based on radiomics, doseomics, and contouromics was shown to be better than the performance of models based on only a single type of omics. In addition, combining traditional radiomics features with other features (such as deep learning features or genetic features) and combining different imaging methods (such as combining CT imaging and PET imaging) have revealed new pathways in radiomics research.

Ensuring the interpretability of a radiomics model is the last step prior to the model’s clinical application. Some studies have suggested that a model can be explained from a global or local perspective. LIME is used to build simple models to explain the local parts of a complex model; PDP and global interpretation techniques, such as ALE, are used to visualize complex models; and interpretation methods, such as SHAP and gene masking techniques, are used to quantify the relationship between features and diseases (or genes) and thereby increase the interpretability of a model.

### Limitations and suggestions

Although the studies reviewed above have demonstrated that radiomics achieves good results when applied to tumors, many limitations hinder the broad application of radiomics models in real-world clinical settings. Some studies have attempted to alleviate these limitations, but current technology is not sufficiently advanced to completely eliminate them. Given these challenges, we now offer some insights and suggestions.

#### Feature selection

A large number of radiomics features can be derived from images in radiomics study. It is important to control the number of radiomics features because too many features can lead to overfitting. To the best of our knowledge, so far, there is no absolute rule that fits all scenarios to determine the number of radiomics features required for modeling. In radiomics-based study, the optimal number of features is often determined by cross-validation on the internal validation data. This cross-validation strategy is applicable to any scenario because it is training data-based. However, studies do show that there is a relationship between the number of features and the number of training samples through a large number of experiments. For example, Hua et al. [187] selected 7 classifiers and carried out extensive experiments to find the relationship between the number of features and the training sample size. They found that the behavior of the optimal-feature-size relative to the sample size depends strongly on the classifier and the feature-label distribution. An immediate corollary is that one should be wary of rules-of-thumb generalized from specific cases. In addition, the performance of a designed classifier can be greatly influenced by the number of features and therefore one should attempt to use a number close to the optimal number. This means that it can be useful to refer to a database of optimal-feature-size curves to choose a feature size, even if this means making a necessarily very coarse approximation of the distribution model from the data—even perhaps just a visual assessment of the data.

#### Feature reproducibility

Many studies have explored the reproducibility of features in different situations, but it appears that no studies have systematically summarized features that are robust to various influencing factors. Most studies have focused on image segmentation when exploring the reproducibility of features. However, the reproducibility of manual segmentation is usually not high due to large inter-expert deviations in defined tumor boundaries. The features obtained by some automatic or semi-automatic segmentation algorithms are more robust than manual features, but manual segmentation has been the most common form of segmentation conducted in recent clinical research. This may be because researchers have paid more attention to Acc in small-scale studies, whereas radiomics results have been applied in clinical practice. The reproducibility of segmentation may be more important than its Acc, as was noted by Kumar et al. [188]. Moreover, even if manual segmentation is replaced by automatic segmentation in the future, this does not mean that the same type of automatic segmentation will be applicable in all situations. Thus, it may be necessary to select an appropriate automatic segmentation algorithm for a given research application and set of parameters. Similarly, no consensus has been reached on the optimal ICC threshold, but it is often the preferred indicator for reproducibility analysis. Therefore, it may be apposite for future research to identify an appropriate ICC threshold value.

Test–retest analysis is often conducted for dimensionality reduction and selection of robust radiomics features with minimal changes, but test–retest analysis results may not be generalizable, and it is recommended that conditions specific to treatment sites and time, scanners, and imaging protocols are used [189]. However, test–retest may be impractical as its utility may not be understood or accepted by patients in a clinic. The phantom study is not described too much in this article for feature stability and repeatability studies. Phantom study can improve the reproducibility and robustness of radiomics features, while the phantom can also be used to accurately measure multicenter differences between different scanners or environments. The reproducibility of radiomics features based on CT is affected by material-dependence [190], which means that it is very important to select appropriate phantom materials according to the characteristics of different tumor sites and ensure that the range of features value between them is similar. Although phantom studies have shown promising trends in relevant radiomics analyses, it remains doubtful whether the phantom study results can be transferred to clinical studies. Mackin et al. [191] partially answered this question; they found that the variability in the values of radiomics features in phantom CT images was comparable to the variability in the values of radiomics features in NSCLC tumor CT images.

#### Model generalizability

Poor model generalizability is one of the main reasons why models have failed to be widely adopted in clinical practice. Methods that have been used to improve generalizability are discussed in this study, and studies have revealed that no single method is applicable to all scenarios. It is not enough to extract robust features to improve model generalizability. Oliveira et al. [192] stated that it is necessary to use standardized multicenter datasets for radiomics research, and they found that a model constructed based on a standardized multicenter dataset had better predictive performance (AUC: 0.67–0.74) than a predictive model constructed based on robust features in validation cohorts (AUC = 0.53). Nevertheless, another study [193] came to the opposite conclusion: the features of multicenter MR images exhibited significantly more variations than those of single-center MR images. Most of the variations were related to the differences in hardware and acquisition, which can influence apparent diffusion coefficient diagrams. The authors provided ways to correct the data variations, such as by discarding poorly reproducible features, performing normalization, and using statistical models that specifically take center effects into account. The authors also mentioned that the reproducibility of high-order radiomics features was poor, which suggests that researchers should carefully select high-order features when modeling. Furthermore, technologies such as harmonization or few-shot learning are not optimal solutions; even though researchers try their best to improve generalizability, the resulting models cannot be perfectly applied to all target datasets. To sum up, to improve model performance on external validation datasets, the solutions can be organized into three categories: data-level, structure-level and algorithm-level. At the data-level, multicenter data standardizing can be used to reduce the distribution difference between training data and external validation data. In structure-level, centralized or distributed learning structure can make multicenter studies close to single-center studies. At algorithm-level, transfer learning can leverage knowledge from target-related domains to train a model on a target task. Manifold learning aims to use regularization terms to minimize the distribution differences between training data and external validation data. In real application scenarios, to the best of our knowledge, so far, there is no absolute rule for determining which strategy can be used to improve model performance on external validation datasets. With the development of federated learning, perhaps structure-level will become more favored.

Radiomics features are highly sensitive to various parameters, and every step in the radiomics workflow must be considered when using additional methods to attempt to improve the stability features, which increases the cost of radiomics analysis. Therefore, data sharing and pipeline standardization are the only way to reduce the variation in radiomics data and improve the generalizability of models. However, it remains difficult to standardize radiomics workflows and implement these at all institutions. Therefore, a model’s generalizability should be improved as much as possible, and then the model should be externally validated to improve its reliability so that it can be applied to clinical decision making.

#### Imbalanced data classification

Imbalanced datasets are common in learning models based on tumor images (as hospitals or institutions store too few samples of target patients), and this is a major limitation of radiomics. This can be addressed by two types of sampling methods, as summarized in a study [194] that contrasted these methods’ effects on radiomics model performance with that of classifier tuning methods and feature selection algorithms. This showed that an optimal choice of classifiers and feature selection algorithms could significantly improve model performance but did not show that sampling methods had a great impact on model performance. Thus, algorithm optimization methods are better than sampling methods in this context. However, Xie et al. [195] argued that model parameter tuning and feature selection did not significantly improve the Acc of a radiomics model. Instead, they found that over-sampling techniques such as ADASYN and SMOTE can raise the geometric means and F-measures of minority class data of HNC patients, thereby improving the predictive performance of imbalanced datasets. Therefore, we propose that to maximize the performance of a model, the best modeling method for the given research dataset must be comprehensively determined.

#### Multi-modality fusion

As multi-modality fusion has always been an obstacle in radiomics, and multi-modality-based models often generate better results than single-modality-based models, multi-modality fusion has strong potential for improving radiomics models and is a current research hotspot. If imbalanced classes are present in a data set, a classifier is biased towards the majority class when learning and thus generates incorrect predictions, which affects the robustness of the model. This is a difficult problem that must be solved, and two types of solutions have been devised: data-based improvements and algorithm-based improvements. Multi-modality can be a combination of radiomics features with other types of features or radiomics with other omics, or a combination of multiple imaging methods, and refinement can result in a combination of different MR imaging sequences [196]. It is well understood that multi-modality methods can compensate for the shortcomings of single-modality methods. Early fusion can use different types of data for complementary purposes. For example, the preferred imaging method for tumors is CT, but this has insufficient soft-tissue contrast; however, the addition of MRI or PET information to a CT data set can overcome this problem to some extent [197]. Late fusion improves final results by fusing models’ results. It has the obvious advantage that model errors do not affect each other and thus errors do not accumulate. Intermediate fusion can perform multiple fusions between layers, which further explores the complementarity between modalities that is explored via early fusion. In summary, this explains why multi-factor models are generally superior to single-factor models. Thus, when conducting fusion research, researchers should perform numerous experiments with different settings and predictive models to enable careful selection of fusion modality and timing, as this will enhance the quality of the resulting model. In reviewing the literature, we found that even though the number of studies using multi-modality fusion has increased in recent years, it remains a rare approach overall and requires further attention from researchers.

#### Model interpretability

Radiomics, due to its high complexity and use of black-box machine learning, has the problem of low interpretability in both its features and models. This problem has caused physicians and specialists to distrust radiomics and has been the biggest obstacle to the widespread adoption of radiomics models in clinical settings. Researchers have thus developed methods to increase the interpretability of radiomics features and models. At the feature level, exploring the association between features and tumor heterogeneity can increase interpretability. At the model level, diverse technologies based on local and global interpretation can be applied to improve the interpretability of a model, although such technologies have their own shortcomings. For example, a feature map formed by deep convolutional neural networks can allow the most uninterpretable deep learning fields to realize the visual interpretation of classification tasks based on these networks [198]. However, we found that not all of the techniques for interpreting black-box models have been thoroughly studied in the field of radiomics, so this remains a task for future research. In addition, as most of the learning of radiomics models is retrospective and not invariably persuasive, it remains insufficient to use only interpretative models, as no model is applicable to all clinical decision-making scenarios. Researchers must continue to perform prospective studies to verify the clinical utility of the methods described in this review.

## Conclusions

In this review, we introduce the radiomics-based studies in cancers from the perspectives of clinical applications and AI-driven modeling. In the first perspective, we provide a particular focus on three distinct applications: tumor grading, tumor staging, and the classification of benign vs. malignant tumors. In the second perspective, we devote more text to discussing feature engineering and statistical modeling in AI-driven radiomics modeling, including feature reproducibility, feature interpretability, model generalizability, model interpretability, imbalanced data classification, and multi-modality fusion. Our comprehensive review reveals that, in the context of AI, radiomics-based studies indeed play an important role in the diagnosis and prognostic prediction of cancers. However, related studies on feature reproducibility, feature interpretability, model generalization and model interpretability still present challenges, which are obstacles to the further promotion of radiomics models to clinical real-world applications.

## Data Availability

Not applicable.

## Abbreviations

Acc: Accuracy
ACM: All-cause mortality
AdaBoost: Adaptive boosting
ADASYN: Adaptive synthetic-nominal
AI: Artificial intelligence
AJCC: American Joint Committee on Cancer
ALE: Accumulated local effects
ANN: Artificial neural network
ANOVA: Analysis of variance
AUC: Area under the receiver operating characteristic curve
CCC: Concordance correlation coefficient
CNN: Convolutional neural network
COV: Coefficients of variation
CT: Computed tomography
CV: Cross validation
DFS: Disease-free survival
DL: Deep learning
DM: Distant metastasis
DT: Decision tree
ENE: Extra-nodal extension
fILD: Fibrosing interstitial lung disease
GLCM: Grey level co-occurrence matrix
GTV: Gross tumor volumes
HCC: Hepatocellular carcinoma
HNC: Head and neck cancer
HNSCC: Head and neck squamous cell carcinoma
HPV: Human papillomavirus
IC: Induction chemotherapy
ICC: Intraclass correlation coefficients
KNN: K-nearest neighbors
KPCA: Kernel principal component analysis
LASSO: Least absolute shrinkage and selection operator
LIME: Local interpretable model-agnostic explanations
LNM: Lymph node metastasis
LOOCV: Leave one out cross validation
LR: Logistic regression
MI: Mutual information
ML: Machine learning
MRI: Magnetic resonance imaging
mRMR: Minimal redundancy maximal relevance
MTV: Metabolic tumor volume
NAC: Neoadjuvant chemotherapy
NCA: Neighborhood component analysis
NPC: Nasopharyngeal carcinoma
NSCLC: Non-small cell lung cancer
OPC: Oropharyngeal cancer
OPSCC: Oropharyngeal squamous cell carcinoma
OS: Overall survival
PCA: Principal component analysis
pCR: Pathologic complete response
PDP: Partial dependence plots
PET: Positron emission tomography
PFS: Progression-free survival
PTV: Planning target volumes
QUS: Quantitative US
RA: Rim average
RF: Random forest
RFE: Recursive feature elimination
ROI: Region of interest
RUSBoost: Random under-sampling and AdaBoost combination model
SHAP: Shapley additive explanations
SM: Statistical method
SMOTE: Synthetic minority over-sampling technique
SPL: Solitary pulmonary lesions
SVM: Support-vector machine
US: Ultrasonography
VT: Variance-threshold
XGBoost: Extreme gradient boosting
2D: Two-dimensional
3D: Three-dimensional
